# A novel TM4SF4-targeting therapeutic antibody candidate with antitumor activity by blocking IGF1R and CD44 signaling and downregulating PD-L1 and B7-H4

**DOI:** 10.7150/thno.113347

**Published:** 2026-01-01

**Authors:** Rae-Kwon Kim, Chang-Kyu Heo, Mun Ju Choi, Yeon-jee Kahm, Min Kyu Kim, Minyong Lee, Ji Yoon Lee, Hwangseo Park, Uhee Jung, Byung-Chul Shin, Bum-Jin Kim, Sung-Chul Kim, Eun-Wie Cho, Chun Jeih Ryu, In-Gyu Kim

**Affiliations:** 1 Department of Radiation Biology, Environmental Radiation Research Group, Korea Atomic Energy Research Institute (KAERI), Daejeon 34057, Republic of Korea.; 2 Department of Biotechnology, Yong In University, 134, Yongindaehak-ro, Cheoin-gu, Yongin-si, Gyeonggi-do, 17092, Republic of Korea.; 3 Infectious Disease Research Center, Korea Research Institute of Bioscience and Biotechnology (KRIBB), Daejeon 34141, Republic of Korea.; 4 Department of Integrative Bioscience and Biotechnology, Institute of Bioscience, Institute of anticancer medicine development, Sejong University, Seoul 05006, Republic of Korea.; 5 Department of Radiation Life Science, Korea University of Science and Technology (UST), Daejeon 34113, Republic of Korea.; 6 Algok Bio Inc. 320 120th Ave. NE #208 Bellevue, WA 98005, USA.

**Keywords:** TM4SF4, EMT-associated stemness, monoclonal antibody (2B7), humanized antibody (Hz2B7-1.2), signaling blocking

## Abstract

**Rationale:** Transmembrane 4 superfamily member 4 (TM4SF4) has been identified as a key regulator of epithelial-mesenchymal transition (EMT)-associated stemness in non-small cell lung cancer (NSCLC) cells through autocrine signaling involving insulin-like growth factor 1 (IGF1) and osteopontin (OPN). Given its pivotal role in tumor progression and therapy resistance, TM4SF4 represents a promising therapeutic target.

**Methods:** To develop a therapeutic antibody against TM4SF4, we generated anti-TM4SF4 monoclonal antibodies in mice by targeting the large extracellular loop (LEL) of human TM4SF4 using a 15-mer peptide, hTM4SF4 (T126-E140). Among the generated clones, the 2B7 antibody exhibited high specificity and reactivity to TM4SF4. Mechanistic studies were conducted to evaluate the effects of 2B7 on key signaling pathways, EMT-associated stemness, immune checkpoint ligand (ICL) expression, and immune responses. To facilitate clinical translation, 2B7 was humanized, generating the Hz2B7-1.1 antibody, which underwent affinity maturation to select the lead candidate, Hz2B7-1.2. Functional assays, including antibody-dependent cellular cytotoxicity (ADCC) and preclinical evaluations in xenograft models, were performed to assess its therapeutic potential.

**Results:** The 2B7 antibody demonstrated significant antitumor efficacy in both A549 xenograft and patient-derived xenograft (PDX) models. Mechanistically, 2B7 inhibited key signaling pathways, including PI3K/AKT/GSK3β/β-catenin and JAK2/STAT3, leading to a reduction in EMT-associated stemness and therapy resistance. Additionally, 2B7 downregulated the expression of ICLs, such as PD-L1 and B7-H4, promoting T-cell activation and mitigating immune evasion. Furthermore, 2B7 reduced the secretion of exosomal ICLs by tumor cells and enhanced antitumor immune responses. The humanized antibody Hz2B7-1.2 retained binding properties and antitumor activity comparable to the parental 2B7 antibody and effectively induced ADCC as an IgG1-type antibody.

**Conclusions:** The humanized anti-TM4SF4 antibody, Hz2B7-1.2, exhibits strong antitumor activity through multiple mechanisms, including inhibition of oncogenic signaling pathways, reduction of EMT-associated stemness, and modulation of immune responses. These findings support Hz2B7-1.2 as a promising therapeutic candidate for TM4SF4-positive cancers, warranting further clinical investigation.

## Introduction

Lung cancer is the most common cancer and the leading cause of cancer-related deaths worldwide 1. Non-small cell lung cancer (NSCLC) accounts for over 75% of all lung cancer cases and remains one of the most difficult to treat because of its high resistance to conventional therapies such as chemotherapy, γ-radiation, and targeted therapy 2,3. Recently, immunotherapy using immune checkpoint inhibitors (ICIs) has significantly improved the outcomes of NSCLC, extending survival in approximately 20% of patients 4. However, resistance to immunotherapy has become a major challenge, necessitating new strategies to overcome disease progression 4,5. A comprehensive understanding of the mechanisms underlying therapy resistance—including resistance to immunotherapy—and poor prognosis in NSCLC is essential for the development of effective treatments 6.

PD-L1 (B7-H1) is a transmembrane immune checkpoint ligand that is expressed in cancer and hematopoietic cells. By binding to the PD-1 receptor on immune cells such as T cells, PD-L1 suppresses immune activation and promotes cancer immune evasion 7. PD-L1 is frequently upregulated in various cancers, including triple-negative breast cancer and NSCLC 8,9. Similarly, B7-H4, another B7 family ligand, negatively regulates T-cell responses and is overexpressed in cancers, including lung cancer, correlating with poor outcomes 10,11. Therefore, targeting B7 ligands or their receptors is a promising strategy to overcome T cell anergy and restore antitumor immunity. Antibodies against PD-1, CTLA-4, and PD-L1—collectively known as ICIs—have demonstrated substantial efficacy in treating cancers, such as melanoma and lung cancer 12,13, while therapies targeting B7-H4 are entering clinical trials 14. To further enhance ICI efficacy, it is important to understand the regulatory mechanisms governing PD-L1 and B7-H4 expression in cancer cells.

Cancer stem cells (CSCs) play a critical role in shaping the tumor immune microenvironment and may contribute to ICI resistance 15,16. Epithelial-to-mesenchymal transition (EMT), which is closely linked to increased expression of immune checkpoint molecules, represents another potential mechanism of immune escape 17. Conversely, mesenchymal-to-epithelial transition (MET) has been proposed as a strategy to sensitize tumors to ICIs 18. Thus, targeting CSCs and modulating EMT/MET dynamics may improve ICI efficacy by overcoming immune resistance mechanisms 19.

The tetraspanin TM4SF family plays a crucial role in signaling pathways that regulate cell growth, migration, and development 20. Among its members, TM4SF1 and TM4SF5 are frequently overexpressed in various tumors, including hepatocellular carcinoma (HCC) and prostate cancer, where they promote EMT-associated signaling and are regarded as important therapeutic targets 21-23. TM4SF4, another member of the TM4SF family, is overexpressed in HCC and colorectal cancer 24,25. Suppression of TM4SF4 significantly inhibits HCC cell growth, underscoring its potential as a target for preventing tumor progression 26. In our previous study using lung adenocarcinoma cells, we found that overexpressed TM4SF4 recruits IGF1R, leading to activation of IGF1Rβ and downstream PI3K/AKT/GSK3β/β-catenin pathway, which is correlated with poor patient prognosis 27,28. TM4SF4 overexpression also enhances the autocrine feedback loop of osteopontin (OPN) and insulin-like growth factor 1 (IGF1), ligands that activate the CD44 and IGF1Rβ signaling pathways 28. Alongside IGF1/IGF1R signaling, OPN/CD44 binding activates JAK2 (or FAK)/STAT3 pathways, promoting EMT-associated CSC properties, tumor metastasis, and γ-radiation resistance 28,29. TM4SF4 is also overexpressed in ALDH1^high^ CSC-like cells 28. These observations suggest that TM4SF4 is a promising molecular target, particularly in therapy-resistant CSC-like cancers.

Monoclonal antibodies (mAbs) serve as both diagnostic and therapeutic agents owing to their high target specificity and stability in vivo 30. Since the introduction of the first therapeutic mAb in 1986 31, mAb development has rapidly progressed, with over 183 approved products and more than 1,200 in clinical development (see: https://www.antibodysociety.org/resources/approved-antibodies) 32. While murine mAbs are easy to produce, their therapeutic use in humans is limited by the human anti-mouse antibody (HAMA) response, which reduces efficacy and increases immunogenicity 33. To address this, humanized mAbs were developed by grafting murine complementarity-determining regions (CDRs) onto a human antibody framework in a process known as CDR grafting 34. However, simple CDR grafting can reduce affinity, as some framework region (FR) residues directly interact with the antigen or stabilize the CDR loop conformation 35. Affinity maturation is commonly implemented to optimize therapeutic mAbs, enhancing their binding kinetics and affinity 36,37, usually by introducing targeted mutations in CDR or FR residues informed by the structure of the antigen-antibody complex 38.

In this study, we developed a murine monoclonal antibody targeting human TM4SF4 (anti-hTM4SF4 mAb), designated 2B7, which exhibited potent anti-cancer effects in NSCLC both *in vitro* and *in vivo*. We demonstrated that the anti-cancer efficacy of 2B7 is mediated by the inhibition of the IGF1/IGF1Rβ and OPN/CD44 signaling, both central to cancer stem cell regulation, and by suppressing immune evasion via downregulation of PD-L1 and B7-H4. To enable clinical application of the highly active murine 2B7 antibody, we generated a humanized version through CDR grafting and affinity maturation, and evaluated its therapeutic potential as an anti-cancer agent. Finally, we discuss the future development and clinical application of humanized 2B7 antibodies.

## Methods

### Chemicals and antibodies

All chemicals used were of reagent grade or higher. The antibodies used for western blotting and immunofluorescence assays are listed in Table S1.

### Cell culture and transfections

HEK293T (CRL-3216), A549 (CCL-185), and THLE-2 (CRL-2706) cells were obtained from ATCC, while Calu-3, Huh7, and MIA PaCa-2 cells were from KCLB. Human primary hepatocytes were purchased from Thermo Fisher Scientific. Cells were cultured in DMEM or RPMI-1640 medium (Biowest) with 10% FBS (VWR) and antibiotic-antimycotic solution (Welgene) at 37°C, 5% CO₂, unless otherwise specified. THLE-2 cells were cultured in BEBM medium (Lonza) with SingleQuots Supplement Pack (excluding gentamycin/ amphotericin and epinephrine) supplemented with 5 ng/mL EGF, 70 ng/mL phosphoethanolamine. Murine hybridomas were cultured in DMEM supplemented with HT medium (Sigma-Aldrich). DHFR-deficient CHO-DG44 cells 39 were maintained in DMEM/F12 (Welgene) with HT medium. For DHFR⁺ selection, CHO-DG44 cells were cultured in IMDM (Welgene) with 100 μg/mL G418 (Duchefa). The hTM4SF4 (NM_004617.4) gene was cloned into pEGFP-C2 or p3xFLAG-CMV 7.1 vectors and transfected into HEK293T cells using Lipofectamine 2000 (Invitrogen). TM4SF4-targeting siRNA (5′-CACCUUUCCCAAGAGAUCU-3′) and negative control siRNA (Invitrogen) were transfected using Lipofectamine RNAiMAX (Invitrogen). Cells were analyzed 48 h post-transfection.

### Production of murine anti-hTM4SF4 monoclonal antibodies

Monoclonal antibodies (mAbs) targeting LEL of hTM4SF4 (UniProt: P48230) were generated using a synthetic peptide hTM4SF4 (T126-E140; TWGYPFHDGDYLNDE) modified with an N-terminal cysteine and conjugated to BSA via sulfo-SMCC (Thermo Fisher Scientific). BALB/c mice were immunized, and hybridomas were produced following AbClon (Korea) protocols. Anti-hTM4SF4 hybridomas were screened by ELISA, and the antibodies were purified using protein G chromatography (Cytiva). Purity was confirmed by SDS-PAGE, followed by Coomassie Blue staining.

### cDNA synthesis and CDR sequencing of the 2B7 antibody

Total RNA was extracted from 2B7 hybridoma cells using RNAiso Plus (TaKaRa) and cDNA was synthesized using PrimeScript™ RT Master Mix (TaKaRa). Antibody variable region cDNAs were amplified using modified primers based on a previously reported method 40. For amplification, 5′ MH1/MH2 and 3′ IgG1 primers were used for the heavy chain, while 5′ MK and 3′ Cκ primers were used for the light chain. The amplified cDNAs were digested, subcloned into pBluescript KS(+) and sequenced (Bionics).

### Chimeric and humanized 2B7 antibody generation and characterization

The chimeric 2B7 antibody was generated by fusing variable heavy (VH) and variable kappa (Vκ) region DNA sequences with murine signal peptides via recombinant PCR (primers: Table S2) and subcloning into the pdCMV-dhfr vector 41, yielding pdCMV-dhfr-Chi2B7. For antibody humanization, BLAST analysis identified 3QRG_H and 3QRG_L as the most homologous human frameworks to the 2B7 VH and Vκ regions 42. 2B7 CDRs were grafted onto these frameworks via DNA synthesis. The humanized VH gene (Hz2B7-1.0) was generated by recombinant PCR (primers: Table S3), while the humanized Vκ gene (Hz2B7-0.1) was chemically synthesized (Bionics). Both genes were subcloned into pdCMV-dhfr, yielding the Hz2B7-1.1 expression vector. To enhance the antigen-binding activity of the Hz2B7-1.1 antibody, affinity maturation was performed using a docking simulation model of the antigen-antibody complex. Specific residues in the Hz2B7-1.1 heavy and light chains were mutated via recombinant PCR with synthetic oligonucleotides (Table S4). The resulting expression vectors were transiently transfected into HEK293T cells using polyethyleneimine (Polyscience) and TOM medium (Welgene). Antibodies were purified from culture supernatants via protein G chromatography and analyzed by SDS-PAGE and western blotting with anti-human IgG-HRP antibodies (Thermo Fisher Scientific).

### Homology modeling and docking simulation of the 2B7 antibody

Because the 3D structure of the 2B7 antibody was unavailable, homology modeling was performed using the X836 antibody (PDB: 3MBX) as a template 43. Sequence alignment was conducted using ClustalW 44 and the structural model was built using MODELLER 45. Molecular dynamics (MD) simulations were employed to refine the model, with loop regions reconstructed via enumeration algorithms to account for structural flexibility 46. The hTM4SF4 (T126-E140) peptide structure was optimized through energy minimization. The homology-modeled 2B7 antibody structure served as the receptor model for epitope interaction simulations. Docking simulations within the 2B7 CDR were conducted using AutoDock 47 to evaluate the binding affinity and interaction modes. A modified binding energy function incorporating a ligand dehydration term was applied to improve interaction estimation 48. Among the 20 epitope conformations generated, the lowest binding free energy conformation was selected as the final binding mode.

### Generation of high-producing antibody clones

CHO-DG44 cells were transfected with PvuI-digested antibody expression vectors using Lipofectamine 2000 and TOM™ medium. After 48 h, cells were seeded into 96-well plates (7×10³ cells/well) with selection medium (IMDM, 10% FBS, 100 μg/mL G418). Neomycin-resistant clones were screened by sandwich ELISA after three weeks, and high-producing clones were expanded in 24-well plates under 0.02 μM methotrexate (MTX; Sigma-Aldrich) selection. The selection was repeated with 0.08 μM MTX. Finally, high-producing cells were expanded, and antibodies were purified via protein G chromatography.

### Indirect ELISA

hTM4SF4 (T126-E140)-conjugated BSA (50 ng/well or 100 ng/well) was coated onto Maxisorp 96-well plates (Thermo Fisher Scientific). After blocking with 1% BSA in TBST (TBS containing 0.05% Tween-20), the plates were incubated with primary antibodies for 2 h at 37°C. HRP-conjugated anti-mouse IgG antibody (CST) or HRP-conjugated goat α-human IgG-kappa chain antibody (Bethyl) were used as the secondary reagents. The reaction was developed using Ultra-TMB solution (Thermo Fisher Scientific) or OPD substrate (Sigma-Aldrich), and absorbance was measured at 450 nm or 490 nm using a microplate reader.

### Western blot analysis and immunoprecipitation

Cell extracts were prepared using RIPA buffer (PBS, pH 7.4, with 1.0% NP-40, 0.1% SDS, and 0.5% sodium deoxycholate) supplemented with phosphatase and protease inhibitors (Roche). For immunoprecipitation (IP), 500 µg of RIPA lysates from p3xFLAG-TM4SF4-transfected cells was incubated with specific antibodies at 4°C for 16 h, followed by Protein G bead capture. For western blot (WB), cell lysates or immunoprecipitates were separated by SDS-PAGE, transferred to PVDF membranes, blocked with 10% non-fat milk in TBST, and incubated with primary antibodies overnight at 4°C. HRP-linked secondary antibodies were applied, and signals were detected using an ECL HRP substrate.

### Fluorescence microscopy

Transfected HEK293T or A549 cells were cultured on glass coverslips placed in 35 mm culture plates, fixed with 4% paraformaldehyde (PFA) (Sigma-Aldrich), and incubated overnight at 4°C with specific antibodies in TBS (pH 7.4). Fluorescent staining was performed using either rhodamine-conjugated anti-mouse IgG+IgM+IgA antibody (Abcam) or Alexa Fluor 488-conjugated anti-rabbit IgG antibody (Invitrogen). Nuclei were counterstained with DAPI, and fluorescence images were acquired using a Zeiss LSM510 Meta confocal microscope (Carl Zeiss MicroImaging GmbH).

### Surface Plasmon resonance (SPR) analysis

Binding affinity of anti-TM4SF4 antibodies to the hTM4SF4 (T126-E140) peptide was evaluated using a Biacore T200 system (Cytiva). A biotinylated hTM4SF4 peptide containing a GSAGGS spacer was immobilized on a streptavidin-coated sensor chip (Cytiva). Antibodies were prepared in a two-fold serial dilution series (128-1 nM or 512-16 nM) using HBS-EP+ buffer (Cytiva). SPR measurements were performed at 25°C with a flow rate of 30 μL/min, and affinity constants were determined using Biacore T200 Evaluation Software 3.1 with a 1:1 binding model.

### Tumor cell-based assays

For colony formation assays, 1 × 10³ cells were seeded in 35-mm dishes and treated with 2B7 antibody (5 μg/mL) or control IgG with or without γ-radiation (6 Gy). After 10-14 days, colonies were stained with crystal violet and counted. For sphere formation assays, 1-2 cells were plated in ultra-low attachment 96-well plates (Corning) and cultured in DMEM/F12 (Invitrogen) with growth factors, N-2, and B-27. 2B7 antibody (5 μg/mL) or control IgG was added to the culture, and sphere formation was quantified after 10-14 days. For invasion and migration assays, 2 × 10⁵ cells in serum-free medium with 2B7 antibody or control IgG were seeded into Transwell inserts. Matrigel®-coated inserts were used for invasion assays, while uncoated inserts were used for migration assays. The lower chamber was filled with RPMI-1640 medium with 10% FBS. After 24 h, migrated or invaded cells were stained and counted. For wound healing assays, A549 cells were grown to 80% confluence, scratched, and treated with 2B7 antibody or control IgG (5 μg/mL). Wound closure was assessed after 24 h and calculated as: wound closure (%) = (1 - specific time wound width/initial wound width) × 100%.

### Exosome analysis

A549 cells were cultured in RPMI medium until they reached 70-80% confluence. After washing with PBS, and incubated with exosome-free FBS medium (System Biosciences). After 48 h, conditioned medium was collected, and exosomes were isolated (System Biosciences), resuspended in PBS, quantified, and analyzed via exosome ELISA. For ELISA, mouse anti-CD63 antibody (200 ng/well) was coated onto Maxisorp 96-well plates and blocked with a protein-free blocking buffer (Thermo Fisher Scientific). Exosomes (100 µg) were added and incubated for 2 h at 37°C, followed by primary antibodies (anti-B7-H4, anti-PD-L1, or biotinylated anti-CD63) for 90 min. Detection was performed using HRP-conjugated anti-rabbit IgG antibody or HRP-conjugated streptavidin (Sigma-Aldrich), and the reaction was visualized using Ultra-TMB solution.

### Flow cytometry

Cells were dissociated using TrypLE™ Express Enzyme (Gibco), filtered through 40 μm strainers, and fixed with 4% PFA for 15 min at 4°C. After washing with PBA buffer (1% BSA, 0.01% NaN₃ in PBS, pH 7.4), cells were incubated with mouse, chimeric, or humanized primary antibodies for 1 h at 4°C, followed by incubation with FITC-conjugated secondary antibodies for 30 min. For live-cell analysis, cells were stained with propidium iodide (PI; Sigma-Aldrich) and PI-negative cells were analyzed. Flow cytometry was performed using a FACSCalibur system (BD Biosciences), and the data were analyzed using CellQuest software.

### Measurement of serum stability

Purified antibodies were incubated in 60% human serum at 37°C for up to 4 days. Samples were collected at 0, 3, 24, 48, 72, and 96 h, frozen at -80°C, and analyzed for binding activity to hTM4SF4 peptide-conjugated BSA (0.5 μg/mL) via indirect ELISA. The 0 h sample served as the baseline (100%) for comparison.

### Antibody-dependent cell-mediated cytotoxicity (ADCC) assays

ADCC assays were performed using A549, THLE-2, MIA PaCa-2, and Huh-7 cells with the human FcγRIIIa (F158) Reporter Bioassay (Promega). Target cells were seeded into 96-well plates and incubated for 24 h, and the medium was replaced with ADCC assay buffer. Serially diluted antibodies and effector cells were added at effector-to-target (E:T) ratios of 12:1 or 20:1 and incubated for 6 h. Luminescence was measured using the Bio-Glo luciferase reagent and a GloMax™ luminometer (Promega).

### A549 xenograft and patient-derived xenograft experiments

A549 lung cancer cells (5 × 10^6^) were injected subcutaneously into the right flank of the BALB/c nude mice. When the tumor reached approximately 50 mm³ or 200 mm³, the mice were randomized into control and treatment groups (n = 4 per group). Anti-TM4SF4 antibodies were administered either intratumorally (10 μg per mouse, six doses; total ~3 mg/kg) or intravenously (500 μg per mouse, two doses; total ~50 mg/kg). Control mice received IgG, and tumor growth was monitored every 2-3 days. For the PDX experiments, tumors with high TM4SF4 expression were transplanted into NOD scid gamma (NSG) mice. When tumors reached 100-150 mm³, mice (n = 4 per group) received intraperitoneal injections of 2B7, Hz2B7-1.2, control IgG (500 μg per mouse, two doses; total ~50 mg/kg) or cisplatin (250 μg per mouse, two doses; total ~25 mg/kg), administered at two-day intervals. The study was terminated when control tumors reached 1,000 mm³, with tumor measurements taken three times per week. The tumor volume was calculated using the following formula: (shortest diameter² × longest diameter)/2. All animal experiments were IACUC-approved (KRIBB: KRIBB-AEC-19172, PDX: DNALINK 20-0107-S1A0 (2)). The PDX study was conducted at DNALINK (Korea).

### Immunohistochemistry (IHC) of xenograft tumors

Formalin-fixed xenograft tumor tissues were paraffin-embedded, sectioned, and subjected to hematoxylin and eosin (H&E) staining as well as immunohistochemical staining. For IHC, tissue sections were deparaffinized, rehydrated, and stained with antibodies against CD44, E-cadherin (E-CAD), and vimentin. Visualization was performed using a DAB detection kit (Abcam) according to the manufacturer's protocol. The stained slides were examined under a light microscope at 200× magnification, and representative images were captured. For each sample, at least seven non-overlapping fields of undamaged tumor tissue were selected and imaged. The DAB-stained areas were quantitatively analyzed using ImageJ software to measure staining intensity. Quantification results were statistically compared between the treatment and control groups.

### Pharmacokinetic (PK) analysis in mice

For PK analysis of 2B7 antibody in A549 xenograft mice, 500 µg of 2B7 was administered intravenously on days -5 and 0 when the tumor size reached 50-200 mm^3^. Blood samples were collected at various time points, clotted at 25°C, and centrifuged at 2,000 × g for 15 min at 4°C. The serum was then separated and stored at -80°C. Serum levels of the murine 2B7 antibody were determined by indirect ELISA using a standard curve. For PK analysis of Hz2B7-1.2 in BALB/c nude mice, Hz2B7-1.2 was administered via intraperitoneal injection to 5-week-old female BALB/c nude mice at a dose of 500 µg per injection on Day -2 and Day 0. Blood samples were collected at various time points, clotted at 25°C, and centrifuged at 2,000 × g for 15 min at 4°C. The serum was separated and stored at -80°C. The serum level of the Hz2B7-1.2 antibody was determined by human IgG ELISA using a standard curve. All animal procedures were conducted in accordance with the guidelines of the IACUC at Sejong Univ. (Approval No.: SJ-20220512).

### Statistics and reproducibility

Statistical analyses were performed using GraphPad Prism, version 10.0. Data are presented as the mean ± SEM from at least three independent experiments. Comparisons among groups were made using Student's t-test or one-way ANOVA, with *p* < 0.05 considered significant.

## Results

### Anti-hTM4SF4 mAbs were generated to target the LEL of hTM4SF4

**Figure 1:**
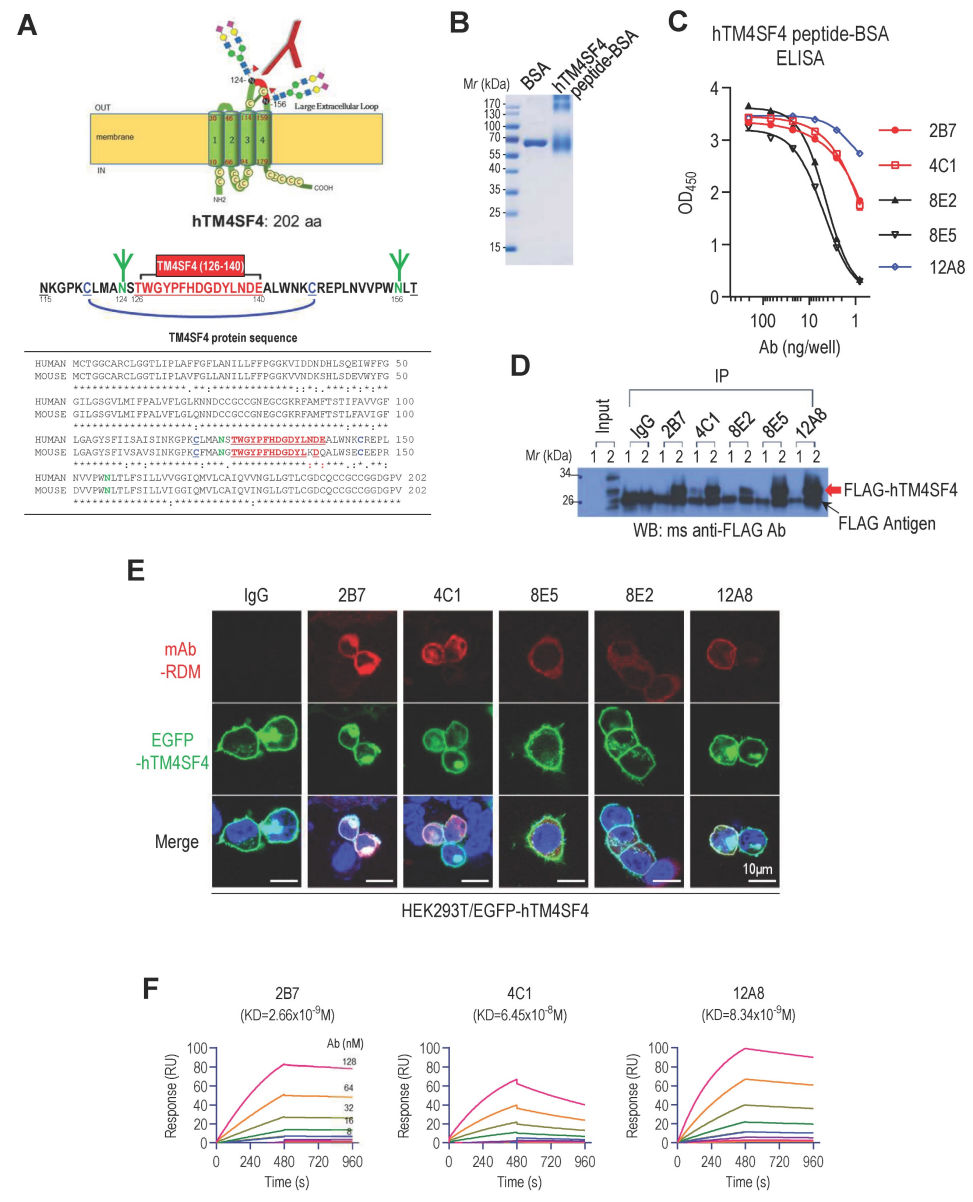
Development of Anti-hTM4SF4 Monoclonal Antibodies Using a 15-Mer Peptide Derived from the hTM4SF4 Extracellular Domain. **A,** Schematic representation of hTM4SF4 within the cell membrane, highlighting the selected antigenic epitope (T126-E140) in red within the LEL. **B,** The synthetic peptide hTM4SF4 (T126-E140) was conjugated to BSA using a Sulfo-SMCC crosslinker. Conjugation was confirmed via 10% SDS-PAGE under reducing conditions. **C,** ELISA results showing the binding affinity of five selected B-cell hybridoma clones, derived from mice immunized with the BSA-conjugated hTM4SF4 peptide. **D,** Immunoprecipitation of FLAG-tagged hTM4SF4 using the novel anti-hTM4SF4 monoclonal antibodies. HEK293T cells lysates transfected with FLAG-hTM4SF4 were immunoprecipitated with the antibodies, followed by western blot analysis with an anti-FLAG antibody. Mouse IgG was used as the control. **E,** Immunofluorescence analysis of hTM4SF4 expression in HEK293T cells using the novel antibodies. HEK293T cells transfected with EGFP-hTM4SF4 were subjected to immunofluorescence staining using the novel anti-hTM4SF4 antibodies, followed by a rhodamine-conjugated secondary antibody (top images). Merged images with EGFP-hTM4SF4 (middle images) show that the novel antibodies can access cell surface TM4SF4. Nuclei were counterstained with DAPI (blue). Scale bar, 10 μm. **F,** SPR sensorgrams showing the concentration-dependent binding kinetics of anti-hTM4SF4 antibodies to the hTM4SF4 peptide. The biotinylated peptide was immobilized on an SA sensor chip, and serially decreasing concentrations of the antibodies (128-1 nM) were applied. The equilibrium dissociation constants (K_D_) are shown in each plot. Detailed kinetic parameters are provided in Table S6.

We aimed to develop mAbs that bind to the extracellular region of TM4SF4 to inhibit its function and suppress cancer stemness in NSCLC. TM4SF4, a transmembrane protein consisting of 202 amino acids, has two extracellular loops, a small loop (SEL; amino acids 31-45) and a large loop (LEL; amino acids 115-158) 49. The SEL domain forms a small loop structure consisting of 15 amino acids, whereas the LEL domain forms a unique loop structure through disulfide bonds between two cysteine residues (C120 and C146) and two N-linked glycans at N124 and N156 (**Figure 1A**). When the TM4SF4 protein sequence was analyzed using the Kolaskar and Tongaonkar method 50, six epitope regions with high antigenicity were identified: amino acids 4-33, 53-67, 71-80, 92-116, 127-133, and 146-198. Among these, only 127-133 sequence is located within the extracellular loop, although partially masked by two N-glycan branches at N124 and N156. Considering the structural features, including N-glycosylation and disulfide bonds, the T126-E140 sequence was selected as the optimal epitope. It is centrally positioned within the C120-C146 loop, encompassing the predicted antigenic site (W127-D133), while avoiding the glycosylation site at N124 (**Figure 1A**). The selected 15-mer peptide, hTM4SF4 (T126-E140), was synthesized and conjugated to BSA using an SMCC crosslinker (**Figure 1B**). Splenocytes from mice immunized with the hTM4SF4 peptide-BSA conjugates were fused with myeloma cells to generate B-cell hybridomas. Screening of the hybridoma by ELISA using the immunizing antigen identified 50 hybridoma clones that produced antigen-specific antibodies. These hTM4SF4-specific clones were further evaluated by comparing their reactivity with hTM4SF4 peptide-conjugated BSA and BSA-SMCC (Figure S1A). This process identified approximately ten epitope-specific clones, which were further refined through repeated limiting dilution assays, ultimately yielding five stable clones: 2B7, 4C1, 8E2, 8E5, and 12A8. The isotypes of these antibodies were determined as IgG1, IgG2a, or IgG2b (Table S5). The antibodies were purified from the cell culture supernatant and analyzed using SDS-PAGE to confirm their purity (Figure S1B). The binding affinity of the purified antibodies to antigenic peptides was further assessed by ELISA using serial dilutions of the antibodies (**Figure 1C**). Among the five tested antibodies, 12A8 exhibited the highest reactivity for the hTM4SF4 peptide​, followed by 2B7 and 4C1, whereas 8E2 and 8E5 showed lower reactivity. All antibodies successfully immunoprecipitated FLAG-tagged TM4SF4 expressed in HEK293T cells, confirming their reactivity with cellular TM4SF4 (**Figure 1D;** Figure S1C). Immunofluorescence analysis further demonstrated that the novel antibodies specifically recognized the extracellular domain of TM4SF4 on the cell membrane.

When EGFP-tagged TM4SF4 was expressed in HEK293T cells, probing with the novel antibodies followed by detection with rhodamine-conjugated secondary antibodies revealed colocalized fluorescent signals on the cellular membranes (**Figure 1E,** lower panel). Notably, stronger fluorescence signals were observed with antibodies 2B7 and 4C1, indicating their higher affinity for, or accessibility to, the target molecule. To confirm the binding of TM4SF4 to the surface of cancer cells, A549 cells were stained with novel antibodies. 2B7, 4C1, and 12A8 successfully stained A549 cells, but failed to stain TM4SF4-knockdown A549 cells, demonstrating their binding specificity to TM4SF4 (Figure S1D). The binding affinities of the anti-hTM4SF4 mAb to the hTM4SF4 peptide were measured by SPR analysis. All three mAbs exhibited nanomolar binding affinities, indicating strong interactions with the epitope peptide, with 2B7 exhibiting the highest binding affinity (**Figure 1F**). Specifically, the equilibrium dissociation constants (KD) for 2B7, 4C1, and 12A8 were 2.66 × 10⁻⁹ M, 6.45 × 10⁻⁸ M, and 8.34 × 10⁻⁹ M, respectively (**Figure 1F**; Table S6).

### Anti-hTM4SF4 mAbs effectively inhibit NSCLC growth *in vitro* and *in vivo*

**Figure 2:**
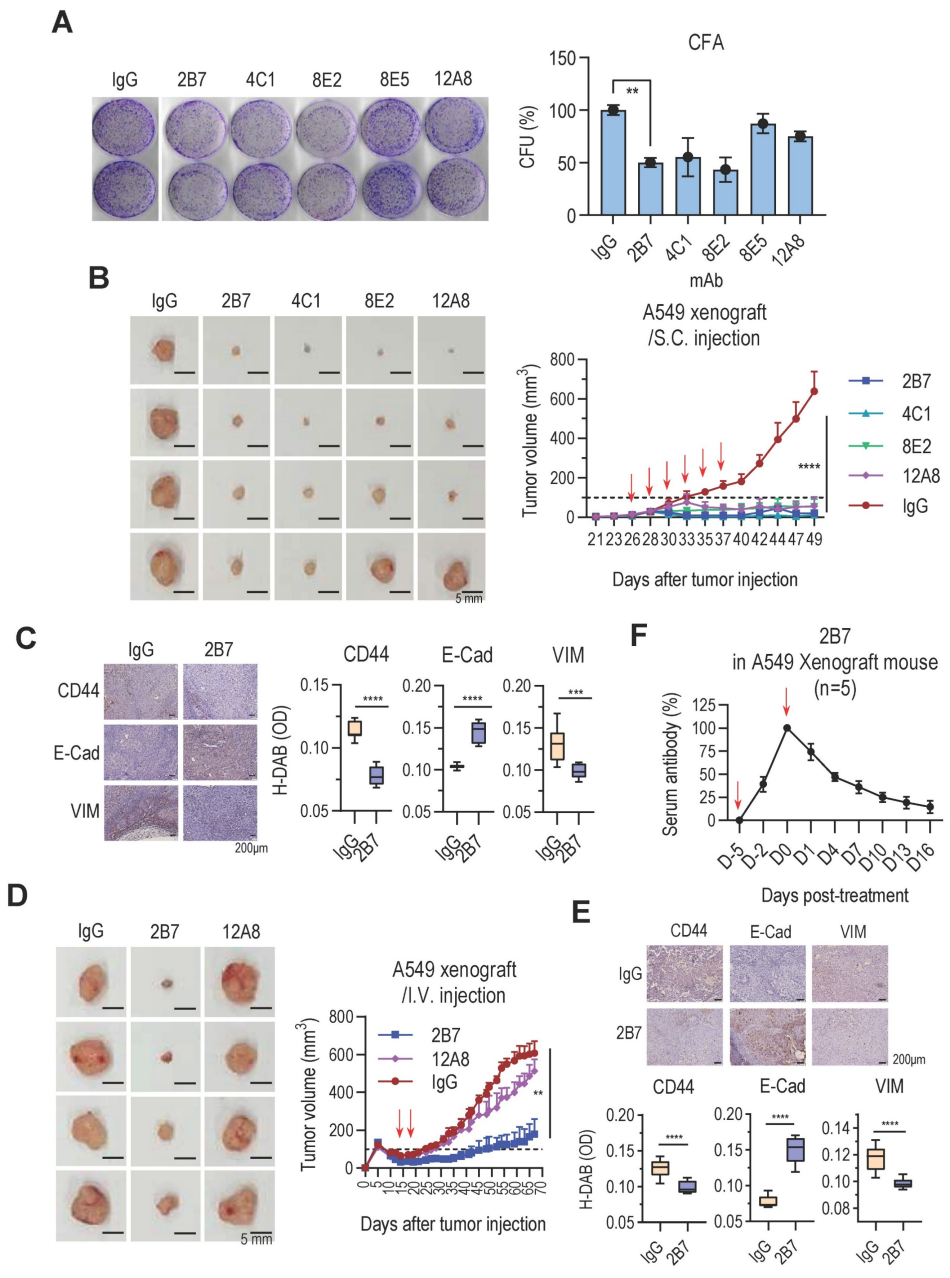
Novel Anti-hTM4SF4 mAbs Inhibit NSCLC Growth both In Vitro and In Vivo. **A,** Colony formation assay showing the inhibitory effects of anti-hTM4SF4 mAbs (2B7, 4C1, 8E2, 8E5, and 12A8) on A549 cells. Cells (3 × 10³ per well) were treated with 5 μg/mL of each antibody. Colony-forming units (CFUs) were quantified relative to control wells treated with mouse IgG. **B,** Subcutaneous administration of anti-hTM4SF4 mAbs (2B7, 4C1, 8E2, and 12A8) inhibited tumor growth in an A549 NSCLC xenograft mouse model. Treatment commenced when tumor volume reached ~50 mm³. Antibodies were administered subcutaneously (10 µg per mouse per dose, six doses; total 60 µg per mouse) at 2-3-day intervals (red arrows). Tumor volumes were measured every 2-3 days and calculated as described in Methods. Tumors were excised and photographed on day 49. **C & E,** Immunohistochemical analysis of the cancer stem cell (CSC) marker CD44 and the epithelial-mesenchymal transition (EMT) markers E-cadherin and vimentin was performed on tumor tissues from mice injected with 2B7 or control IgG. Stained slides were examined under a light microscope at 200× magnification, and representative images were captured. DAB-stained areas were quantitatively analyzed using ImageJ software, and the results were statistically compared between the treatment and control groups. **D**, Intravenous administration of 2B7 and 12A8 further confirmed tumor growth inhibition. Antibodies were injected twice (500 μg per mouse per dose, total 1 mg per mouse) at 5-day intervals (red arrows). Tumors were excised and photographed at the end of the experiment. Scale bar, 5 mm. *n* = 4 per group. **F,** Serum levels of 2B7 in xenograft mice were quantified by ELISA using anti-TM4SF4 antigen and comparison to a standard curve. 2B7 activity measured 4 hours after injection was set as 100% reference for normalization.

To evaluate the therapeutic potential of anti-hTM4SF4 mAbs against NSCLC, their effects on tumor growth were assessed both *in vitro* and *in vivo*. *In vitro* colony formation assays demonstrated that anti-TM4SF4 mAb inhibited the proliferation of A549 lung cancer cells (**Figure 2A**). Notably, 2B7, 4C1, and 8E2 antibodies reduced cell proliferation by approximately 50%, exhibiting greater potency than 8E5 and 12A8. Xenograft experiments confirmed the antitumor efficacy of these antibodies *in vivo*. When A549 xenograft tumors reached approximately 50 mm³, anti-hTM4SF4 mAbs (2B7, 4C1, 8E2, and 12A8) were administered intratumorally, resulting in a significant reduction in tumor size in all treated mice (**Figure 2B**). Immunohistochemical analysis of tumor tissues injected with 2B7 revealed decreased expression of the cancer stem cell (CSC) marker CD44, as well as changes in the epithelial-mesenchymal transition (EMT) markers E-cadherin and vimentin (**Figure 2C**). A similar tumor reduction was also observed when the antibodies were administered to larger tumors (~200 mm³) (Figure S2A). In a separate xenograft model, 2B7, 4C1, and 12A8 antibodies were administered intravenously (**Figure 2D**; Figure S2B). For systemic administration, the antibody dose was increased approximately 16-fold. Among the tested mAbs, 2B7 and 4C1 significantly reduced tumor size, whereas 12A8 exhibited only marginal effects. In this case as well, tumor tissues from mice injected with 2B7 exhibited significant changes in CD44 and EMT marker expression (**Figure 2E**). When 2B7 antibody was administered intravenously, the number of injections was reduced, and the total antibody dose was split into two administrations. Despite this reduced dosing schedule, tumor growth was effectively suppressed. This finding suggests that the antibody maintained sufficient concentrations in tumor tissue to exert a sustained antitumor effect. To evaluate antibody persistence, serum levels of 2B7 were measured in xenograft mice using an anti-TM4SF4 antibody ELISA (**Figure 2F**). Antibody activity decreased to approximately 50% within one-week post-injection and remained around 15% at 16 days. Nevertheless, tumor growth remained suppressed throughout the observation period, indicating that the antitumor activity of 2B7 was effectively sustained over time.

Collectively, these results demonstrate the significant therapeutic potential of novel anti-hTM4SF4 mAbs in suppressing NSCLC tumor growth both *in vitro* and *in vivo*. Among these, 2B7 and 4C1 exhibited the most potent antitumor effects, highlighting their potential as candidates for the development of targeted therapies against NSCLC. However, given that 2B7 demonstrated superior antigen-binding affinity than 4C1, subsequent experiments were primarily conducted using 2B7.

### Anti-hTM4SF4 mAb, 2B7, inhibits self-renewal and EMT in NSCLC cells

**Figure 3:**
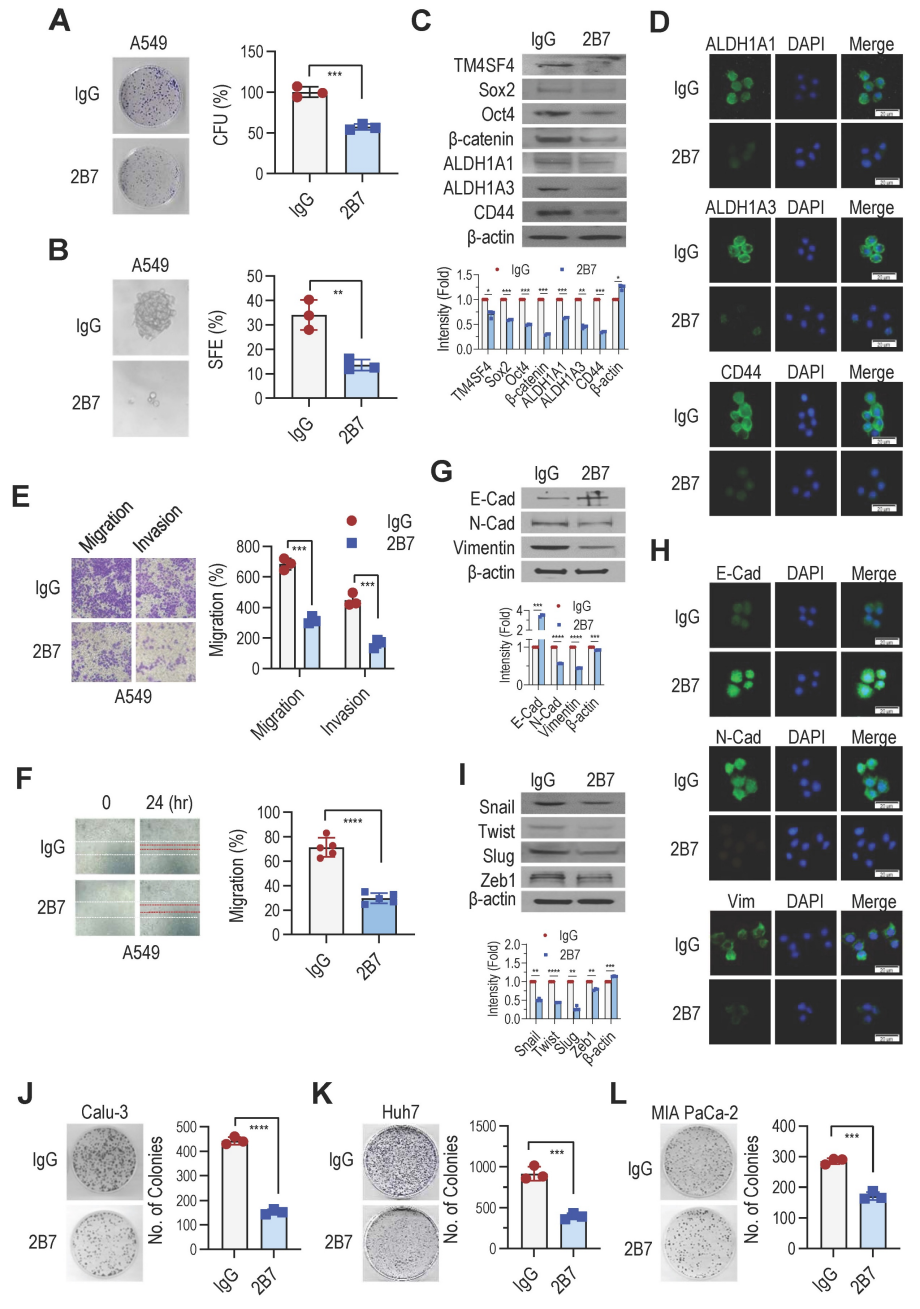
Anti-hTM4SF4 mAb 2B7 Suppresses Cancer Stemness, Migration, and Invasion in A549 NSCLC Cells and Other Cancer Models. **A,** Colony formation assay in A549 cells treated with 2B7 (5 μg/mL). Colonies were stained with crystal violet after 10 days. CFU percentages were calculated relative to mouse IgG controls. **B,** Sphere formation assay in A549 cells treated with 2B7 (5 μg/mL). Spheres were quantified and imaged (400× magnification) after 10-14 days. Sphere-forming efficiency (%) was calculated as spheres formed/total cells plated. **C,** Western blot analysis of CSC markers (Oct4, Sox2, β-catenin, ALDH1A1, ALDH1A3, and CD44) in A549 cells treated with 2B7 (5 μg/mL) for 24 h. Band intensities were quantified using ImageJ. **D,** Immunocytochemistry of CSC markers (ALDH1A1, ALDH1A3, and CD44) in A549 cells treated with 2B7 (5 μg/mL) for 24 h. Scale bar = 20 μm. **E,** Migration and invasion assays of A549 cells treated with 2B7 (5 μg/mL) for 24 h. Migrated/invaded cells were stained and quantified (400× magnification). Percentages were calculated relative to controls. **F,** Wound-healing assay in A549 cells treated with 2B7 (5 μg/mL). Wound closure was imaged at 0 h and 24 h, and migration (%) was calculated as: (1- specific time wound width/initial wound width) × 100%. **G,** Western blot analysis of EMT markers (E-cadherin, N-cadherin, Vimentin) in A549 cells treated with 2B7 (5 μg/mL) for 24 h. **H,** Immunocytochemistry of EMT markers (E-cadherin, N-cadherin, Vimentin) in A549 cells treated with 2B7 (5 μg/mL) for 24 h. **I,** Western blot analysis of additional EMT markers (Snail, Twist, Slug, Zeb1) in A549 cells treated with 2B7 (5 μg/mL) for 24 h. **J-L,** Colony formation assays in Calu-3 (NSCLC), Huh7 (hepatocellular carcinoma) and MIA PaCa-2 (pancreatic cancer) cells treated with 2B7 (5 μg/mL). CFU percentages were calculated relative to controls.

We previously demonstrated that TM4SF4 is associated with CSC properties 28. To determine whether the antitumor efficacy of anti-hTM4SF4 mAb is due to the inhibition of TM4SF4-driven self-renewal, we examined changes in sphere and colony formation abilities following treatment with anti-hTM4SF4 mAbs. Treatment with 2B7 significantly reduced the colony-forming capacity of A549 cells (**Figure 3A**) and markedly inhibited their sphere-forming ability (**Figure 3B**). Additionally, 2B7 treatment decreased the expression of self-renewal markers Sox2, Oct4, and β-catenin, and CSC markers ALDH1A1, ALDH1A3, and CD44 (**Figure 3C**). Immunocytochemistry further confirmed the alterations in the expression of CD44 and ALDH1 following 2B7 treatment (**Figure 3D**).

TM4SF4 has also been implicated in EMT by activating receptor tyrosine kinase (RTK) and integrin-mediated signaling pathways that are critical for EMT, cancer stemness, and angiogenesis 28,51,52. To evaluate whether 2B7 could inhibit TM4SF4-induced EMT, we examined its effects on cell invasion and migration. Treatment with 2B7 significantly reduced the invasion and migration of A549 cells (**Figure 3E**) and impaired cell motility in the wound healing assays (**Figure 3F**). Furthermore, 2B7 treatment increased E-cadherin levels and reduced N-cadherin and vimentin expression, which are key EMT markers (**Figure 3G**). These findings were further validated by immunocytochemical analysis (**Figure 3H**). Additionally, 2B7 treatment reduced the expression of the EMT-regulating transcription factors Snail, Twist, Slug, and Zeb1 (**Figure 3I**). 2B7 also suppressed the growth of another NSCLC cell line, Calu-3 (**Figure 3J**). Beyond NSCLC, 2B7 inhibited the growth of other TM4SF4-expressing cancer cell lines, including Huh7 (hepatocellular carcinoma) and MIA PaCa-2 (pancreatic cancer) (**Figure 3K-L**). These three tumor cell lines express substantial levels of TM4SF4 (Figure S3).

Collectively, these findings demonstrate that the 2B7 antibody, which showed effective antitumor efficacy in NSCLC xenograft models, inhibited TM4SF4-mediated cancer stemness and EMT and provided evidence of its therapeutic potential in extrapulmonary cancer types.

### 2B7 Antibody inhibits feedback autocrine effects of IGF1 and OPN in NSCLC cells

**Figure 4:**
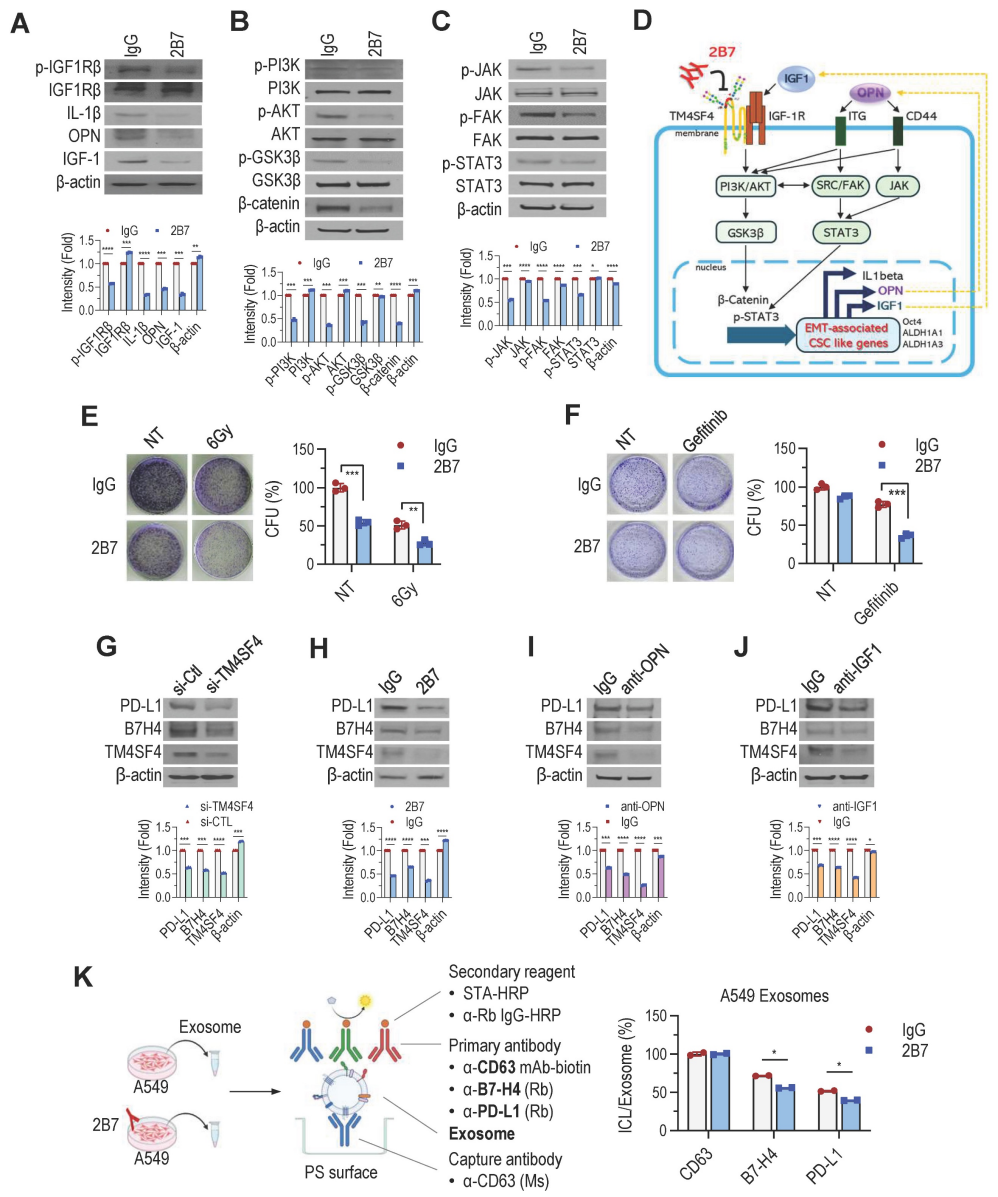
2B7 Inhibits the Feedback Autocrine Effects of IGF1 and OPN, Enhancing the Sensitivity of NSCLC Cells to Antitumor Therapy. **A-D,** Suppression of TM4SF4-associated signaling by 2B7 (5 μg/mL) in A549 cells. **A**, IGF1Rβ phosphorylation and autocrine cytokines (IGF1, OPN, IL-1β) were reduced. **B,** PI3K/AKT/GSK3β pathway (downstream of IGF1R) was inhibited. **C,** JAK (FAK)/STAT3 pathway (downstream of OPN/CD44) was suppressed. **D,** Schematic model depicting 2B7-mediated inhibition of EMT-associated CSC-like properties via disruption of TM4SF4/IGF1 and TM4SF4/OPN autocrine loops and suppression of downstream signaling. **E-F,** Colony formation assays assessing therapy sensitization. **E,** γ-Irradiation (6 Gy) + 2B7 (5 μg/mL). Colony formation was significantly reduced compared to irradiation alone. **F,** Gefitinib (1 μg/mL) + 2B7 (5 μg/mL). Combined treatment showed synergistic inhibition of colony formation. **G-J,** PD-L1 and B7-H4 expression analyses in A549 Cells after treatments. **G,** TM4SF4 knockdown by siRNA (48 h post-transfection). **H,** 2B7 treatment (5 μg/mL, 24 h). **I,** OPN-neutralizing antibody treatment. **J,** IGF1-neutralizing antibody treatment. Mouse IgG was used as the control for all antibody treatments. **K,** Quantification of exosomal PD-L1 and B7-H4 by sandwich ELISA: Tumor-derived exosomes were captured using anti-CD63 (exosome marker). Exosomal PD-L1 and B7-H4 were detected using anti-PD-L1 or anti-B7-H4 rabbit antibodies, followed by anti-rabbit IgG-HRP. Total exosome levels were estimated with anti-human CD63-biotin and streptavidin-HRP. ICL expression in exosomes was presented as the PD-L1/B7-H4 signal ratio relative to total exosome response.

TM4SF4 overexpression in A549 NSCLC cells activates IGF1R, increasing IGF1, OPN, and IL-1β expression 27,28. This leads to IGF1Rβ phosphorylation and downstream activation of the PI3K/AKT/GSK3β and β-catenin pathways. OPN elevation further enhances CD44 activity, promoting EMT-related CSC traits via JAK2/STAT3 and FAK/STAT3 pathways. To elucidate how 2B7 inhibits the cancer cell proliferation, self-renewal, and metastasis, we examined its effects on TM4SF4-related signaling pathways. 2B7 treatment reduced IGF1Rβ phosphorylation, lowering IL-1β, OPN, and IGF1 levels (**Figure 4A**). This disrupted PI3K/AKT/GSK3β and β-catenin signaling (**Figure 4B**). Reduced OPN also diminished CD44 activation, impairing EMT-associated cancer stemness via JAK2/STAT3 and FAK/STAT3 pathways (**Figure 4C**). Overall, these results highlight the potential of 2B7 as a therapeutic antibody for NSCLC by suppressing key cancer stemness and EMT pathways (**Figure 4D**).

Signaling pathways activated by IGF1 and OPN, known to enhance EMT and CSC-like properties, also contribute to the resistance to chemotherapy and radiotherapy 53-55. To assess whether 2B7 could overcome this resistance, we evaluated its effects in combination with other antitumor treatments. A549 cells treated with 2B7 following γ-irradiation showed a synergistic reduction in colony formation compared to controls (**Figure 4E**). Additionally, 2B7 treatment significantly enhanced A549 cell sensitivity to gefitinib, effectively reducing drug resistance (**Figure 4F**). These results suggest that 2B7 is not only effective as a standalone antitumor agent but also enhances therapeutic efficacy when combined with radiotherapy or targeted therapy by suppressing CSC properties and overcoming treatment resistance.

PD-L1(B7-H1) and B7-H4, key ICLs, are overexpressed in tumors, suppressing T-cell activation and facilitating immune evasion 7-9,56,57. B7-H4, abundant in lung cancer, is associated with IGF1R signaling 58, while PD-L1 expression is regulated by OPN from tumor-associated macrophages 59. Our studies revealed that TM4SF4 upregulation enhanced OPN and IGF1 secretion in lung cancer cells 28, suggesting its role in modulating ICL expression. Therefore, targeting TM4SF4 may reduce ICL expression by decreasing extracellular IGF1 and OPN levels, thereby improving the efficiency of cancer therapies. Consistent with this hypothesis, TM4SF4 suppression via siRNA or 2B7 treatment reduced PD-L1 and B7-H4 levels (**Figure 4G-H**). Neutralizing OPN/IGF1 in TM4SF4-overexpressing cells had similar effects (**Figure 4I-J**), with all treatments also reducing TM4SF4 levels. Overall, these results highlight the dual role of 2B7 in inhibiting TM4SF4-mediated cancer stemness and limiting immune evasion by suppressing ICL expression.

Exosomes derived from NSCLC cells are also known to carry PD-L1 and B7-H4, contributing to immunosuppression and tumor progression by further reducing T cell activity 60-62. 2B7 treatment of A549 cells significantly lowered PD-L1 and B7-H4 levels in exosomes, as confirmed by sandwich ELISA (**Figure 4K**). These results indicate that 2B7 not only suppresses ICL expression in cancer cells but also reduces exosomal ICL levels, potentially enhancing antitumor immune responses.

### Humanized 2B7 antibodies were constructed and characterized

As demonstrated, 2B7 exhibits potent antitumor efficacy by targeting TM4SF4. However, murine antibodies face clinical limitations due to their immunogenicity in humans 63. To overcome this challenge and enhance its clinical applicability, the murine 2B7 antibody was engineered into a humanized form, a process that reduces immunogenicity while preserving antigen specificity and therapeutic efficacy.

We first defined the CDR sequence of 2B7 (Figure S4) and constructed an expression vector for the chimeric antibody (Chi2B7) that combines the variable regions of the murine 2B7 antibody with the constant regions of human IgG1 and the κ light chain (Figure S5). Human IgG1, the most commonly used isotype in therapeutic antibodies, is preferred for its ability to induce ADCC and Complement-Dependent Cytotoxicity (CDC). The κ chain was selected for its prevalence and lower immunogenicity. The Chi2B7 expression vector was transfected into HEK293T cells and the recombinant antibody Chi2B7 was purified from culture supernatants (Figure S6A-B). Functional validation showed that Chi2B7 retained the antitumor efficacy of the original murine antibody. ELISA confirmed specific binding of Chi2B7 to the TM4SF4 peptide-BSA, but not to BSA (Figure S6C). Additionally, flow cytometry analysis demonstrated that Chi2B7 effectively recognized and bound to cell surface TM4SF4 in NSCLC cell lines A549 and Calu-3 (Figure S6D).

**Figure 5:**
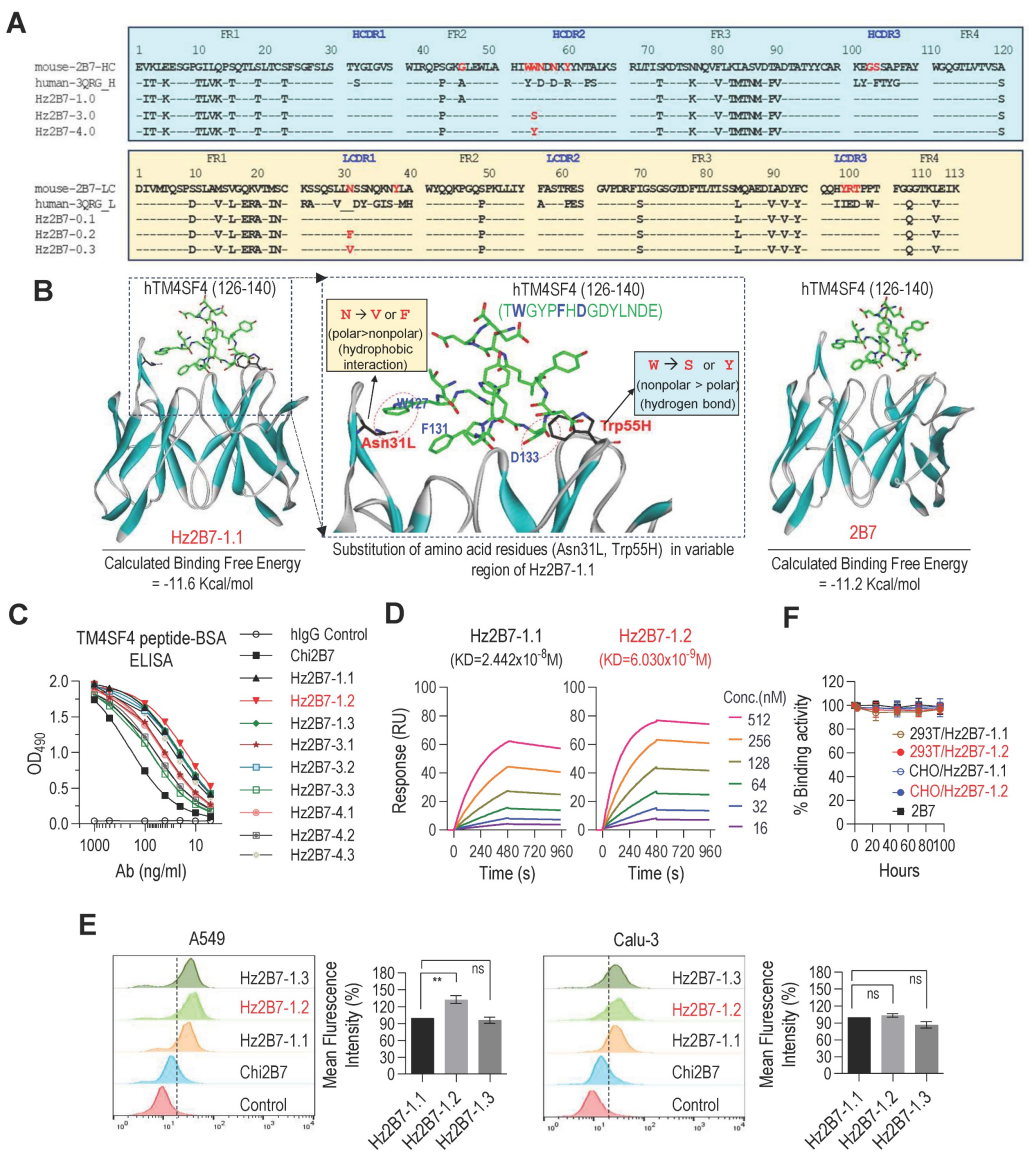
Construction and Characterization of Humanized 2B7 Antibodies. **A,** Comparison of murine 2B7, human 3QRG, and humanized 2B7 antibody sequences. Heavy chain (VH) sequences shown in the upper panel; light chain (Vκ) sequences in the lower panel. Key mutations in the humanized variants are indicated. **B,** Docking Simulation of Epitope-Antibody Interaction: Docked pose of the hTM4SF4 epitope within the CDRs of 2B7 and Hz2B7-1.1. CDRs displayed in ribbon representation; epitope shown as stick models. Dotted circles indicate hydrophobic interactions and hydrogen bonds at the binding interface. **C,** Binding affinity analysis of chimeric and humanized 2B7 antibodies to hTM4SF4 peptide-BSA via indirect ELISA. Hz2B7-1.2 (Vκ N31F) exhibited the highest binding affinity among all tested antibodies. Human IgG served as the negative control. **D,** SPR Analysis of concentration-dependent binding kinetics of humanized 2B7 antibodies to hTM4SF4 peptide. Biotinylated hTM4SF4 (T126-E140) peptide was immobilized on an SA sensor chip, and serially decreasing concentrations of the antibodies (512-16 nM) were applied. KD values were presented on each sensorgram, with detailed kinetics listed in Table S7. **E,** Comparison of binding activity of humanized 2B7 antibodies to NSCLC cell lines (A549 and Calu-3) by flow cytometry. Relative binding was determined based on mean fluorescence intensities obtained from flow cytometric analysis. Data are presented as mean values ± SD (*n* = 3). ns, not significant. **, p < 0.01. **F,** Serum stability of murine 2B7, Hz2B7-1.1, and Hz2B7-1.2 antibodies. Antibodies incubated in 60% human serum at 37°C and evaluated by indirect ELISA for binding activity to hTM4SF4 peptide-BSA. All antibodies produced from CHO or HEK293T cells retained binding activity for up to 96 hours.

To develop a humanized version of the 2B7 antibody, we generated genes encoding the CDRs of the 2B7 antibody grafted onto the framework regions of the human antibody 3QRG, which showed the highest sequence homology to the 2B7 VH and Vκ regions (**Figure 5A**, *see Methods for details*). The resulting humanized 2B7 VH (Hz2B7-1.0) and Vκ (Hz2B7-0.1) genes were subcloned into the pdCMV-dhfr vector, generating the Hz2B7-1.1 expression construct (Figure S5). The pdCMV-dhfr-Hz2B7-1.1 vector was transfected into HEK293T cells, and the humanized antibodies were successfully produced (Figure S7). To enhance the antigen-binding affinity of Hz2B7-1.1, the antigen-antibody binding interface was analyzed in detail using docking simulations. Since the 3D structure of 2B7 has not yet been experimentally determined, homology modeling was performed using the X-ray crystal structure of the chimeric antibody X836 (PDB ID: 3MBX) as a template 43. A structural model of the hTM4SF4 (T126-E140) epitope was generated by energy minimization of the linear peptide. The antigen-antibody interface and binding affinity were then evaluated using the homology-modeled structure of 2B7 and epitope model. As shown in **Figure 5B** (left panel), the peptide epitope fits well within the CDR of Hz2B7-1.1, with a calculated binding free energy of -11.6 kcal/mol, indicating a more thermodynamically favorable interaction compared to the 2B7-peptide complex (-11.2 kcal/mol;** Figure 5B**, right panel) and the 4C1-peptide complex (-10.3 kcal/mol; Figure S8). Based on structural analysis of the Hz2B7-1.1-peptide interface, residue substitutions were explored, targeting positions moderately distant from the epitope to avoid disrupting direct binding interactions. Key residues identified were Asn31L and Thr100L in the light chain (Vκ), and Trp54H, Trp55H, and Asn56H in the heavy chain VH. Notably, Trp55H was positioned at a moderate distance from Asp133 in the epitope. It was hypothesized that substituting Trp55H with hydrogen bond-donating amino acids, such as Ser or Tyr, would strengthen antibody-epitope interactions (**Figure 5B**, middle panel). These substitutions produced two VH domain variants: W55S (Hz2B7-3.0) and W55Y (Hz2B7-4.0) (**Figure 5A,** upper panel). To further enhance the hydrophobic interactions between Hz2B7 and the epitope, Asn31L in the Vκ region was targeted, as its modification could improve interactions with Trp127 and Phe131 of the epitope. Substitution of Asn31L with Val or Phe generated hydrophobic variants: N31V (Hz2B7-0.2) and N31F (Hz2B7-0.3) (**Figure 5A**, lower panel). To further optimize binding affinity, single VH and Vκ mutants were combined to form double mutants: Hz2B7-3.2 and Hz2B7-3.3 (W55S + N31F/N31V); Hz2B7-4.2 and Hz2B7-4.3 (W55Y + N31F/N31V). The resulting pdCMV-dhfr-Hz2B7-1.1 variants were transfected into HEK293T cells for antibody production (Figure S7). Indirect ELISA revealed that all nine Hz2B7 variants specifically bound the TM4SF4 peptide-BSA, outperforming the chimeric Chi2B7 (**Figure 5C**). Hz2B7-1.2 (Vκ N31F) demonstrated the strongest binding activity, followed by Hz2B7-1.3 (Vκ N31V) and Hz2B7-1.1. SPR analysis confirmed these results, with Hz2B7-1.2 showing the highest affinity (K_D_ = 6.03 × 10⁻⁹ M). In contrast, Hz2B7-1.1, chimeric 2B7, and Hz2B7-4.3 displayed K_D_s of 2.442 × 10⁻⁸ M, 6.074 × 10⁻⁸ M, and 8.165 × 10⁻⁸ M, respectively (**Figure 5D**; Figure S9; Table S7). Flow cytometry confirmed that all humanized antibodies had enhanced binding to cell surface TM4SF4 in A549 and Calu-3 cells compared to Chi2B7 (**Figure 5E**). Hz2B7-1.2 showed the strongest binding in A549 cells, outperforming Hz2B7-1.1 and Hz2B7-1.3, though the difference was not significant in Calu-3 cells. Additionally, Hz2B7-1.2 bound to HCC cell lines (Huh7, SNU-387, SNU-449) but not to primary human hepatocytes (hPH) (Figure S10), suggesting potential for clinical application in HCC.

To evaluate *in vitro* serum stability, Hz2B7-1.2 was incubated in 60% human serum at 37 °C (**Figure 5F**). The binding activity remained stable over 96 h, independent of the production cell line (HEK293T or CHO). Comparable stability was observed for Hz2B7-1.1 and the murine 2B7 antibody, indicating that Hz2B7-1.2 is structurally stable in human serum.

### Hz2B7-1.2 exhibits anti-cancer effects and mediates cellular cytotoxicity against TM4SF4-expressing cancer cells

**Figure 6:**
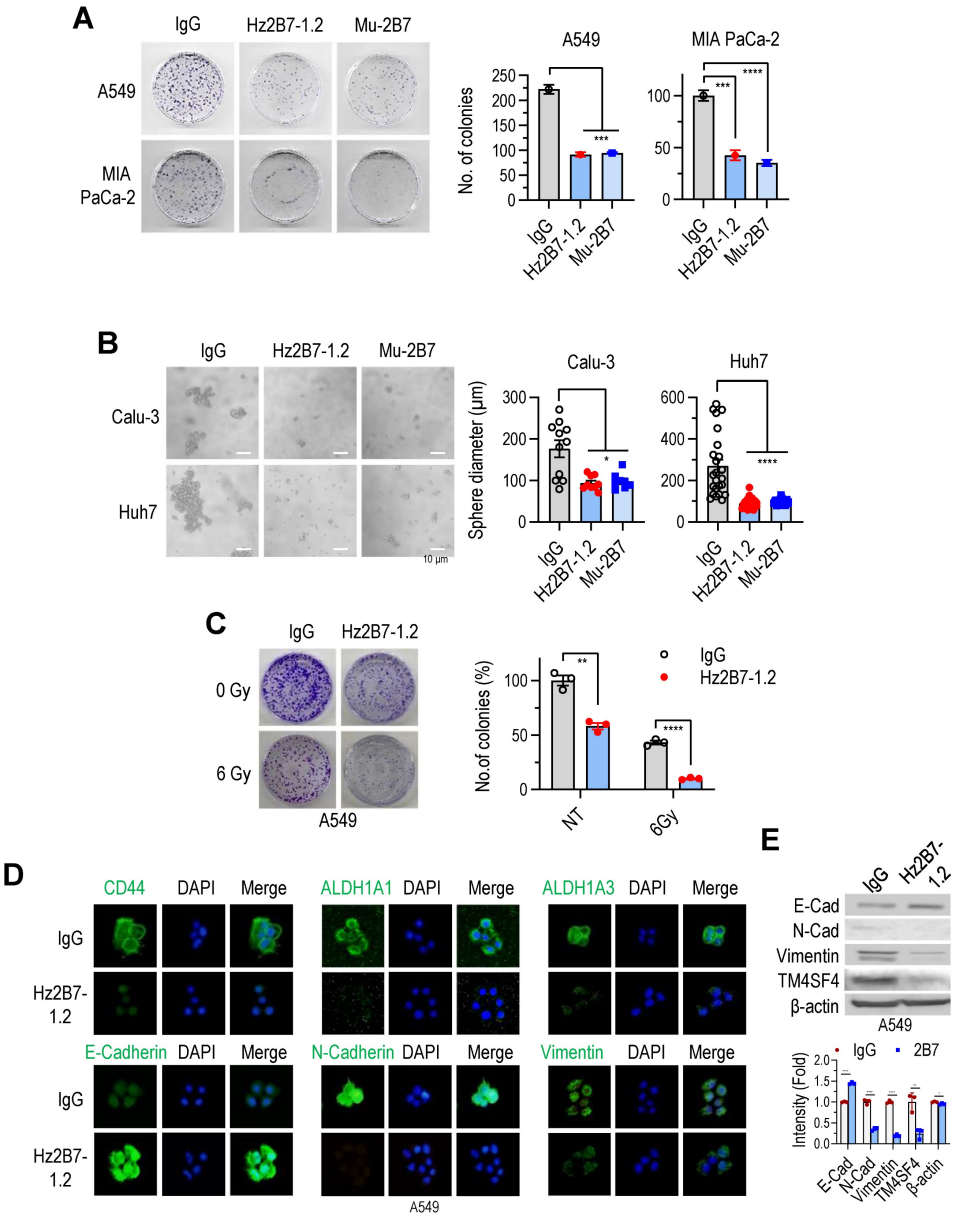
Humanized 2B7 antibody, Hz2B7-1.2 Inhibits Cancer Cell Growth and Self-Renewal**. A,** Colony formation inhibition in A549 and MIA PaCa-2 cancer cells treated with Hz2B7-1.2 (5 μg/mL). Human IgG was used as a control, and murine 2B7 was included for comparison. CFU percentages were calculated relative to the IgG control. **B,** Inhibition of sphere formation in Calu-3 NSCLC cells and Huh-7 HCC cells treated with Hz2B7-1.2 (5 μg/mL). Spheres diameters were measured after 14 days (n ≥ 8). **C,** Radiosensitization effect of Hz2B7-1.2 in A549 cells treated with 5 μg/mL antibody following 6 Gy γ-irradiation. Colony formation was quantified. Data are presented as mean ± SEM. **, p < 0.01; ***, p < 0.001; ****, p < 0.0001. **D,** Immunocytochemical analysis of cancer stemness markers (CD44, ALDH1A1, and ALDH1A3) and EMT markers (E-cadherin, N-cadherin, and Vimentin) in A549 cells treated with Hz2B7-1.2 (5 μg/mL) for 24 h. Scale bar = 20 μm. **E,** Western blot analysis of EMT markers and TM4SF4 expression in Hz2B7-1.2-treated A549 cells. Results were quantified relative to controls.

The antitumor efficacy of the humanized Hz2B7-1.2 antibody was evaluated *in vitro and in vivo*, alongside its murine counterpart, 2B7. In colony formation assays, both antibodies similarly inhibited A549 cell growth at equivalent concentrations (**Figure 6A**). Additionally, both antibodies suppressed the growth of other cancer cell lines, including Calu-3 (NSCLC; Figure S11A), Huh7 (hepatocellular carcinoma; Figure S11A), and MIA PaCa-2 (pancreatic cancer; **Figure 6A**). Treatment with Hz2B7-1.2 in these cells reduced cellular TM4SF4 expression (Figure S11B), indicating effective binding of the antibody to tumor cells. Cell cycle changes were also assessed following treatment with TM4SF4 antibodies. siRNA-mediated knockdown of TM4SF4 resulted in a marked increase in the G0/G1 phase and a corresponding decrease in the S phase (Figure S12A-C). Treatment with the Hz2B7-1.2 antibody also induced cell cycle alterations; although the effect was less pronounced than with siRNA knockdown, a moderate decrease in the S phase was observed (Figure S12D-E).

In spheroid formation assays using Calu-3 (lung cancer) and Huh7 (liver cancer) cell lines, spheroid formation reduced by over 50% following treatment with either antibody (**Figure 6B**). Additionally, Hz2B7-1.2 enhanced the radiosensitivity of A549 cells, showing effects comparable to murine 2B7 (**Figure 6C; Figure 4E**). Hz2B7-1.2 treatment also modulates the expression of CSC- and EMT-related markers. Immunofluorescence analysis showed a reduction in CSC markers (CD44, ALDH1A1, and ALDH1A3) and alterations in EMT markers, including increased E-cadherin and decreased N-cadherin and Vimentin, in response to Hz2B7-1.2 treatment (**Figure 6D**). To quantify the changes in EMT markers and TM4SF4 expression, Western blot analyses were performed and results were quantified relative to the controls (**Figure 6E**).

**Figure 7:**
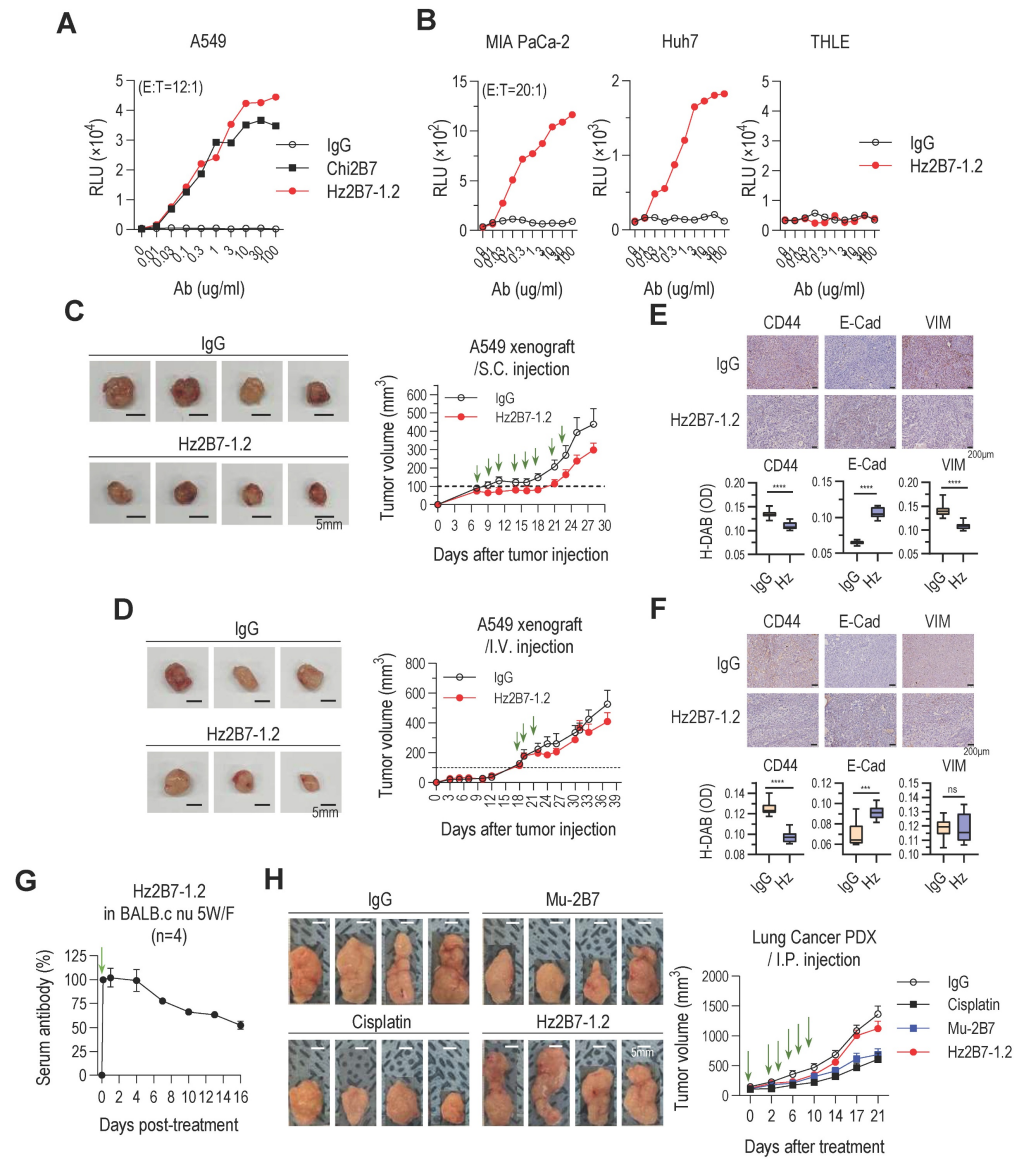
Hz2B7-1.2 Mediates Cellular Cytotoxicity and Inhibits Tumor Growth in TM4SF4-Expressing Tumors.** A,** ADCC activity against A549 cells. The hFcγRIIIa (F158) ADCC reporter assay was performed. Effector cells were co-cultured with A549 NSCLC cells at an E:T ratio of 12:1 in the presence of serially diluted antibodies for 6 h. Luminescence detection was conducted to assess ADCC activity. Human IgG1 was used as a negative control, and Chi2B7 was tested for comparison. **B,** ADCC activity in other cancer cell lines. The ADCC assay was repeated using MIA PaCa-2, Huh7 cancer cells, and THLE-2 non-tumorigenic liver epithelial cells. Effector cells were incubated with target cells (E:T = 20:1) in the presence of serially diluted antibodies for 6 h. Hz2B7-1.2 induced dose-dependent ADCC in cancer cells but showed no cytotoxicity in THLE-2 cells, highlighting tumor specificity. **C,** Tumor growth inhibition in A549 xenograft model after subcutaneous administration. Hz2B7-1.2 (10 µg/mouse/dose) was subcutaneously administered eight times at 2-3 day intervals (total 80 µg/mouse). Treatment started when tumor size reached ~100 mm³. Tumor growth was monitored every 2-3 days, and tumors were excised for imaging at the end of the study. Scale bar = 5 mm; n = 4 per group. **D,** Intravenous administration in A549 xenograft model. Hz2B7-1.2 (500 µg/mouse/dose) was intravenously injected three times at 3-day intervals (total 1.5 mg/mouse). Tumor volume was measured throughout the study. n = 3 per group. **E&F,** Immunohistochemical analysis of CD44, E-cadherin and vimentin was performed on tumor tissues from mice injected with Hz2B7-1.2 or control IgG (E: subcutaneous, F: intravenous injection). Stained slides were examined under a light microscope at 200× magnification, and representative images were captured. DAB-stained areas were quantitatively analyzed using ImageJ software, and results were statistically compared between the treatment and control groups. **G,** PK analysis of Hz2B7-1.2 in mice. 5-week-old female BALB/c nude mice were administered 500 µg Hz2B7-1.2 intraperitoneally on Day -2 and Day 0. Serum samples were collected at specified time points, and Hz2B7-1.2 concentrations were quantified using a human IgG ELISA based on a standard curve. n = 4 per group. **H,** Tumor growth in a PDX model treated with anti-hTM4SF4 antibodies (2B7 and Hz2B7-1.2). High TM4SF4-expressing patient lung tumor tissues were transplanted into NSG mice. Treatments were administered intraperitoneally when the average tumor size reached 100-150 mm³, included anti-hTM4SF4 mAbs (2B7 and Hz2B7-1.2), control IgG, and cisplatin. Each of the treatment groups received six injections at two-day intervals (green arrows). The total dosages were 50 mg/kg for the antibody and 25 mg/kg for cisplatin. Tumor size was measured every four days, and the experiment was terminated when the tumor size in the control group reached 1,000 mm³. n = 4 per group.

Therapeutic antibodies exert antitumor effects through both Fab-mediated target inhibition and Fc-mediated effector functions. A critical mechanism, ADCC, depends on antibody binding to target antigens and the recruitment of cytotoxic immune cells (NK cells, macrophages, monocytes). Trastuzumab (anti-HER2) is a well-established ADCC-inducing antibody 64, while ADCP plays a vital role in the efficacy of antibodies like rituximab and daratumumab 65-67. To assess the ADCC potential of Hz2B7-1.2 (IgG1 isotype) against TM4SF4-positive A549 cells, we performed an ADCC assay using FcγRIIIa (F158)-expressing effector cells. Hz2B7-1.2 induced a dose-dependent ADCC response, effectively activating effector cells (**Figure 7A**). Hz2B7-1.2 exhibited higher ADCC activity than the chimeric Chi2B7 antibody at equivalent doses, likely due to enhanced TM4SF4 binding (Table S7). The control antibody showed no cytotoxicity under the same conditions. Additionally, Hz2B7-1.2 induced robust ADCC in MIA PaCa-2 (pancreatic cancer) and Huh7 (hepatocellular carcinoma) (**Figure 7B**), but not in THLE-2 (normal liver cells), confirming its specificity for TM4SF4-expressing cancer cells (Figure S3).

The *in vivo* antitumor efficacy of Hz2B7-1.2, a humanized antibody combining the superior TM4SF4-targeting ability of 2B7 with the effector functions of IgG1, was evaluated. In the A549 xenograft model, intratumoral injections of Hz2B7-1.2 significantly inhibited tumor growth (**Figure 7C**). Intravenous administration of Hz2B7-1.2 also reduced tumor size (**Figure 7D**); however, its antitumor effect was less pronounced than that of the murine 2B7 antibody (**Figure 2D**). Immunohistochemical analysis reflected these results, showing changes in CSC and EMT markers after administration of the humanized antibody; however, these effects were less marked than those observed with the murine antibody (**Figure 7E**-**F**). To determine whether the reduced antitumor efficacy of Hz2B7-1.2 following intravenous injection was related to its *in vivo* instability, we conducted a serum stability assessment in BALB/c mice after intraperitoneal injection. The serum concentration of Hz2B7-1.2 began to decline four days post-injection, reaching ~50% of initial levels by day 16 (**Figure 7G**). The observed half-life and clearance were considered moderate, given that natural IgG1 antibodies typically exhibit a half-life of ~3 weeks and monoclonal IgG1 antibodies display a broad half-life range (6-32 days) 68. Thus, the reduced efficacy of Hz2B7-1.2 is unlikely due to serum instability. To further assess its therapeutic efficacy, Hz2B7-1.2 and murine 2B7 were evaluated in a TM4SF4-expressing lung cancer PDX model (Figure S13). Both antibodies were administered via intraperitoneal injection (total dose of 50 mg/kg), matching the A549 xenograft model. The control group received cisplatin (total dose of 25 mg/kg), close to the lethal threshold. While murine 2B7 achieved tumor suppression comparable to cisplatin, Hz2B7-1.2 led to only a modest tumor size reduction (**Figure 7H**).

Collectively, these results demonstrate that the humanized Hz2B7-1.2 antibody successfully grafted and preserved the superior TM4SF4 target-binding properties of the parental murine 2B7 antibody. Additionally, as an IgG1 isotype, Hz2B7-1.2 effectively mediates antibody-dependent cellular cytotoxicity (ADCC) against TM4SF4-expressing cancer cells, resulting in tumor clearance. However, its antitumor efficacy was significantly lower than that of the murine 2B7 antibody in the tumor mouse model. This reduced efficacy is likely due to structural and functional differences between the murine and humanized antibodies, particularly the effector cell recruitment functions introduced in the IgG1-type humanized antibody, which are absent in the murine IgG1 antibody.

## Discussion

A major challenge in cancer therapy is the selective elimination of cancer cells while sparing normal tissues. To overcome this, targeted therapies have been developed, with monoclonal antibodies playing a pivotal role due to their high specificity for tumor-associated antigens. In particular, target-specific antibodies against molecules such as TM4SF4, which is highly expressed in cancer stem cells, are expected to make significant contributions to antitumor strategies. In this study, we successfully generated monoclonal antibodies against TM4SF4—a transmembrane protein overexpressed in lung cancer cells—through immunization of mice with a short antigenic peptide derived from its LEL domain. Although concerns existed regarding glycan-mediated shielding of the targeted epitope, located between two proximal N-glycan structures, *in vitro* experiments confirmed sufficient antibody accessibility. The central location of the epitope within a loop structure formed by two cysteine residues, with minimal conformational complexity, enabled a short linear peptide to elicit high-affinity antibodies. Importantly, antibody binding to this region effectively modulated TM4SF4 function, highlighting its therapeutic potential.

Among the novel anti-TM4SF4 monoclonal antibodies developed, 2B7—a murine IgG1 antibody with a κ light chain—demonstrated robust antitumor effects in both *in vitro* and *in vivo* lung cancer models. 2B7 markedly inhibited key malignant phenotypes, including cell growth, stemness, migration, invasion, and radiation resistance. Despite the generally low FcγR affinity of murine IgG1 69, 2B7 exhibited potent antitumor efficacy, likely through direct blockade of TM4SF4 signaling, a crucial regulator of cancer stemness. Notably, this study is the first to show that TM4SF4 signaling promotes immune checkpoint ligand (ICL) expression, facilitating immune evasion. Treatment with 2B7 effectively suppressed ICL expression in both cancer cells and exosomes, thereby enhancing antitumor immunity.

To translate 2B7 for clinical use, we engineered a humanized version, Hz2B7-1.2 (human IgG1 subclass), via CDR grafting and affinity maturation. SPR and cell-based assays demonstrated that Hz2B7-1.2 retained antigen-binding affinity after humanization. Serum stability studies showed that Hz2B7-1.2 remained sufficiently stable *in vivo.* However, while *in vitro* efficacy persisted, the *in vivo* antitumor activity of Hz2B7-1.2 was reduced, particularly following systemic administration. Since both 2B7 and Hz2B7-1.2 bind TM4SF4 equivalently *in vitro,* this difference is likely attributable to variations in Fc-mediated interactions.

Murine immune cells can respond to both murine and humanized antibodies, allowing for partial assessment of ADCC and other Fc-dependent functions in mouse models. Human IgG1 antibodies can engage murine Fcγ receptors (FcγRI and FcγRIII) with differing affinities to induce ADCC, whereas murine IgG2a and IgG2b isotypes display even stronger ADCC due to higher FcγRIII affinity 70. In contrast, the murine IgG1 isotype exhibits weaker effector function. In our study, 2B7—due to low FcγR affinity—likely remained predominantly as free antibody in mouse circulation, facilitating direct targeting and high antitumor efficacy. Conversely, Hz2B7-1.2 is expected to interact more efficiently with FcγRs, leading to immune complex formation and reduced free antibody levels, potentially limiting antigen accessibility and antitumor potential (Figure S14). IgG1 antibodies generally have a molecular weight of approximately 150 kDa and a size of 10-14 nm; by comparison, immune complexes formed with immune cells can exceed this size by several orders of magnitude. While sufficient antigen accessibility enables Fc-mediated cytotoxicity by humanized IgG1 antibodies, epitope shielding by glycans may restrict efficacy to only those capable of accessing the target site.

Under *in vitro* settings without immune cells, both 2B7 and Hz2B7-1.2 directly bind the cancer cell antigen, yielding similar effects; in vivo, their engagement with immune cells diverges, explaining differences in efficacy. Murine antibodies 4C1 and 12A8, although similar to 2B7 in antigen binding, showed reduced antitumor efficacy, likely due to their high FcγR affinity that limits free antibody levels for direct targeting.

Enhancing Hz2B7-1.2 efficacy will require preservation of Fab-mediated target binding, reduction of FcγR engagement to maintain higher free antibody levels, and retention of FcRn affinity for optimal serum stability. Potential strategies include Fc glycan modification or switching subclasses to IgG2 or IgG4 71,72, minimizing immune cell interactions as successfully demonstrated in anti-PD-1 therapeutics 73. Future research will focus on such optimization of Hz2B7-1.2 to maximize its clinical potential. Given the demonstrated effectiveness of 2B7 in hepatocellular and pancreatic cancer models, anti-TM4SF4 monoclonal antibody therapy may be broadly applicable to other TM4SF4-overexpressing tumors, supporting the development of universal CSC-targeted cancer therapies.

## Supplementary Material

Supplementary figures and tables.

## Abbreviations

ADCC: antibody-dependent cellular cytotoxicity
CDC: complement-dependent cytotoxicity
CDRs: complementarity-determining regions
CSCs: cancer stem cells
EMT: epithelial-mesenchymal transition
FR: framework region
HAMA: human anti-mouse antibody
ICIs: immune checkpoint inhibitors
ICL: immune checkpoint ligand
IGF1: insulin-like growth factor 1
LEL: large extracellular loop
mAbs: monoclonal antibodies
MET: mesenchymal-to-epithelial transition
MTX: methotrexate
NSCLC: non-small cell lung cancer
OPN: osteopontin
PDX: patient-derived xenograft
SEL: small extracellular loop
SPR: surface plasmon resonance
TM4SF4: transmembrane 4 superfamily member 4
