# Supplementary material for: A novel TM4SF4-targeting therapeutic antibody candidate with antitumor activity by blocking IGF1R and CD44 signaling and downregulating PD-L1 and B7-H4
Source: Theranostics. 2026 Jan 1;16(4):1762–81. doi: 10.7150/thno.113347 (PMC12680531; doi:10.7150/thno.113347)
Supplement: Supplementary file 1 — Supplementary figures and tables. [file thnov16p1762s1.pdf]

Table S1. List of Antibodies Used in the Present Study

Primary antibody	Catalog number	Company
**FLAG-tag**	MA1-91878	Thermo Fisher Scientific
**hTM4SF4**	NBP1-69647	Novus Biologicals
**Sox2**	#3579	Cell Signaling Technology (CST)
**Oct4**	#2750	CST
**β-catenin**	#8480	CST
**ALDH1A1**	ab6192	Abcam
**ALDH1A3**	ab129815	Abcam
**CD44**	#3570	CST
**β-actin**	sc-47778	Santa Cruz, Biotechnology
**E-cadherin**	ab15148	Abcam
**N-cadherin**	#610920	BD Transduction
**Vimentin**	MA5-14564	Invitrogen
**Snail**	sc-10432	Santa Cruz
**Twist**	sc-15393	Santa Cruz
**Slug**	sc-166476	Santa Cruz
**Zeb1**	sc-25388	Santa Cruz
**Phospho-IGF1R**	sc-81499	Santa Cruz
**IGF1Rβ**	sc-3027	Santa Cruz
**IL-1β**	AF-401-NA	R&D system
**Osteopontin**	AF1433	R&D system
**IGF1**	AF-291-NA	Bio-Techne
**Phospho-PI3K**	sc-293115	Santa Cruz
**PI3K**	#3011	CST
**Phospho-AKT**	#9271	CST
**AKT**	#9272	CST
**Phospho-GSK3β**	#9322	CST
**GSK3β**	#9315	CST
**Phospho-JAK**	#3711	CST
**JAK**	#3230	CST
**Phospho-FAK**	#8556	CST
**FAK**	#3285	CST
**Phospho-STAT3**	#9145	CST
**STAT3**	sc-8019	Santa Cruz
**PD-L1**	#13684	CST
**B7-H4**	12080-1-AP	Proteintech
**CD63**	ab8219	Abcam
**CD63-biotin**	ab134331	Abcam
**Normal mouse IgG1**	sc-3877	Santa Cruz

Table S2. Primers Used for Recombinant PCR of the Chimeric 2B7 Antibody Gene Cloning

Gene		Primer Sequences (5'→3')
Heavy chain signal peptide	Forward	GAC GAA TTC ACT CTA ACC ATG GAA TGG A
	Reverse	CTT CAC CTC GGA GTG GAC ACC TGT AGT TA
Heavy chain variable region	Forward	GTC CAC TCC GAG GTG AAG CTG GAG GAG TC
	Reverse	TTG GGC CCT TGG TGG AGG CTG CAG AGA CAG TGA CCA G
Light chain signal peptide	Forward	CTG CAA AGC TTC GGC ACG AGC A
	Reverse	CAC AAT ATC TCC TTC AAC ACC AGA CAA CC
Light chain variable region	Forward	GTT GAA GGA GAT ATT GTG ATG ACC CAG TCT
	Reverse	CCA CCG TAC GTT TGA TTT CCA GCT T

Table S3. Primers Used Recombinant PCR to Produce a Gene Sequence Encoding the Heavy Chain Variable Region, Referred to as Hz2B7-1.0

Primer	Sequence (5`→ 3`)
2B7-S-H-5`	GAC GAA TTC ACT CTA ACC ATG GAA TGG AGC TGG GTC TTT CTC TTC TTC CTG TCA GTA ACT ACA GGT GTC CAC TCC GAG ATC ACC CTG AA
2B7-S-H-3`	CTC AGG CTG AAG CCG CTG AAG GTG CAG GTC AGG GTC AGG GTC TGG GTG GGC TTC ACC AGG GTG GGG CCG CTC TCC TTC AGG GTG ATC TCG GAG
2B7-HC-1-5`	CAG CGG CTT CAG CCT GAG CAC TTA TGG TAT AGG AGT AAG CTG GAT CAG GCA GCC CCC CGG CAA GGC CCT GGA GTG GCT GGC CCA CAT TTG
2B7-HC-1-3`	CCT GGT TCT TGC TGG TGT CCT TGG TGA TGG TCA GCC TGC TCT TCA GGG CTG TGT TAT AGT ACT TAT TAT CAT TCC ACC AAA TGT GGG CCA GCC AC
2B7-HC-2-5`	ACA CCA GCA AGA ACC AGG TGG TGC TGA CCA TGA CCA ACA TGG ACC CCG TGG ACA CCG CCA CCT ACT ACT GCG CCA GGA AGG AGG GCA GCT
2B7-HC-2-3`	GGG CCC TTG GTG GAG GCG CTG CTC ACG GTC ACC AGG GTG CCC TGG CCC CAG TAA GCA AAG GGG GCC GAG CTG CCC TCC TTC CTG G
Ch57-HC-5`	GAC GAA TTC ACT CTA ACC AT
2B7-WH-3`	TTG GGC CCT TGG TGG AGG CGC TGC T

Table S4. Primers Used for Humanized 2B7 Antibody Affinity Maturation

Gene	Primer	Sequence (5`→ 3`)
Heavy Chain	**h2B7-H.C-W55S-5`**	TTT GGT CGA ATG ATA ATA AGT A
	**h2B7-H.C-W55S-3`**	TCA TTC GAC CAA ATG TGG GCC A
	**h2B7-H.C-W55Y-5`**	TTT GGT ATA ATG ATA ATA AGT A
	**h2B7-H.C-W55Y-3`**	TCA TTA TAC CAA ATG TGG GCC A
	**h2B7-H.C-SLIC-5`**	GCC AGT GTG CTG GAA TTC ACT CTA ACC
	**h2B7-H.C-SLIC-3`**	AAG ACC GAT GGG CCC TTG GTG GAG
Light Chain	**h2B7-L.C-N31F-5`**	TTT TAT TCA GTA GCA ATC AA
	**h2B7-L.C-N31F-3`**	CTA CTG AAT AAA AGG CTC TGA C
	**h2B7-L.C-N31V-5`**	TTT TAG TAA GTA GCA ATC AA
	**h2B7-L.C-N31V-3`**	CTA CTT ACT AAA AGG CTC TGA C
	**h2B7-L.C-SLIC 5`**	ATA GGG AGA CCC AAG CTT CGG CAC GAG CAG A
	**h2B7-L.C-SLIC 3`**	TGG TGC AGC CAC CGT ACG CTT GAT CTC CA

Table S5. Isotypes of Mouse Anti-hTM4SF4 mAbs Generated in This Study

Clones	ELISA* (OD450)	Ig Isotype	
		HC	LC
**2B7**	**3.654**	**IgG1**	Kappa
**4C1**	3.578	**IgG2a**	Kappa
**8E2**	3.686	**IgG1**	Kappa
**8E5**	3.521	**IgG2a**	Kappa
**12A8**	3.685	**IgG2b**	Kappa

*Coating antigen: hTM4SF4 (126-140) peptide-BSA

Table S6. Summary of SPR analysis for Mouse Anti-TM4SF4 Antibodies

mAb	2B7	4C1	12A8
k_a (1/Ms) #	4.85E+04	1.69E+04	2.47E+04
k_d (1/s)	1.29E-04	1.09E-03	2.06E-04
K_D (M) §	**2.66E-09**	**6.45E-08**	**8.34E-09**
Rmax (RU)	103.4	129.5	131.6
χ^2 (RU^2) $	1.47	7.42	0.254
U-value ξ	12	7	1

#Sensor Chip: Biotin-hTM4SF4 (T126–E140) peptide captured SA sensor
§ k_a/k_d model: 1:1 binding mass transfer.
$ χ^2 :< 10% of Rmax is significant for the experimental data
ξThe U-value: an estimate of the uniqueness of the calculated values for rate constants and Rmax. If the U-value is below about 15 the parameter values are not significantly correlated.

Table S7. Summary of the SPR analysis for Humanized 2B7 Antibodies

mAb	Chi-2B7	Hz2B7-1.1	Hz2B7-1.2	Hz2B7-4.3
k_a (1/Ms) #	5.077E+03	7.523E+03	1.259E+04	9.585E+03
k_d (1/s)	3.084E-04	1.837E-04	7.590E-05	7.827E-04
K_D (M) §	6.074E-08	**2.442E-08**	**6.030E-09**	8.165E-08
Rmax (RU)	26.53	76.30	81.32	63.53
χ^2 (RU^2) $	0.0809	0.252	1.78	0.352
U-value	3	2	12	1

#Sensor Chip: Biotin-TM4SF4$_{(126\text{-}140)}$ peptide captured SA sensor
§ k_a/k_d model: 1:1 binding mass transfer.
$ χ^2 :< 10% of Rmax is significant for the experimental data
ξThe U-value: an estimate of the uniqueness of the calculated values for rate constants and Rmax. If the U-value is below about 15 the parameter values are not significantly correlated.

Figure S1

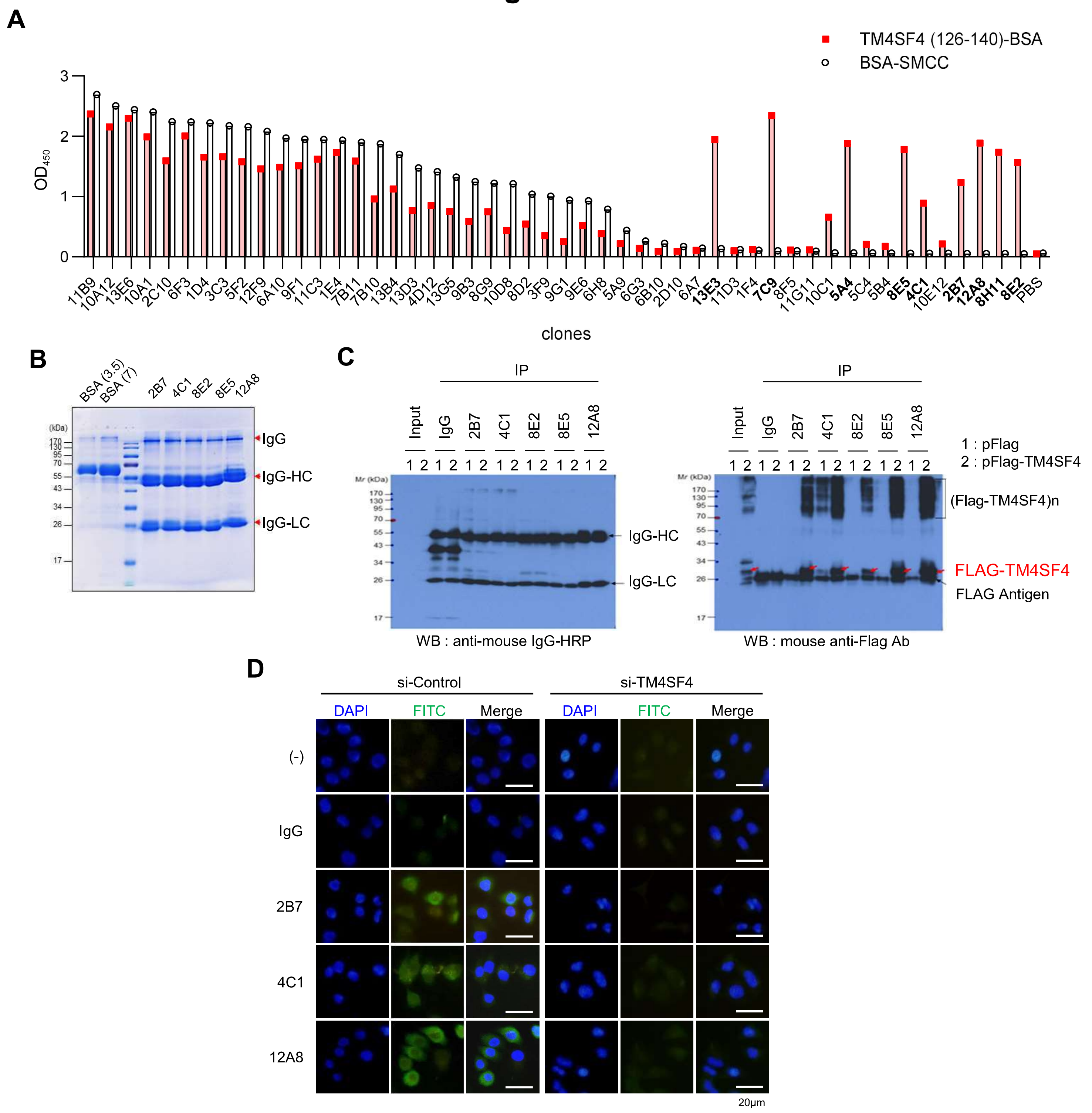

Figure S1. Generation of anti-hTM4SF4 mAbs using a 15-mer peptide derived from the large extracellular loop of hTM4SF4 (A) BALB/c mice were immunized with an hTM4SF4 (126–140) peptide-BSA conjugate for B cell hybridoma generation, which yielded fifty B cell hybridoma clones. The reactivity of antibodies produced by these B cell hybridomas against antigenic peptide were determined by ELISA using the hTM4SF4(126–140) peptide conjugated to BSA. BSA modified with SMCC was used as a control antigen. **(B)** Five antibodies against hTM4SF4(126–140) were purified and analysed by 10% reducing SDS-PAGE. **(C)** Five novel anti-hTM4SF4 antibodies immunoprecipitated the FLAG tagged TM4SF4 expressed in HEK293T cells (original data of Figure 1D). **(D)** A549 cells were stained with anti-hTM4SF4 mAbs (2B7, 4C1 and 12A8) and an FITC-conjugated secondary reagent, whereas TM4SF4-knockdown suppressed FITC staining with these antibodies.

Figure S2

A

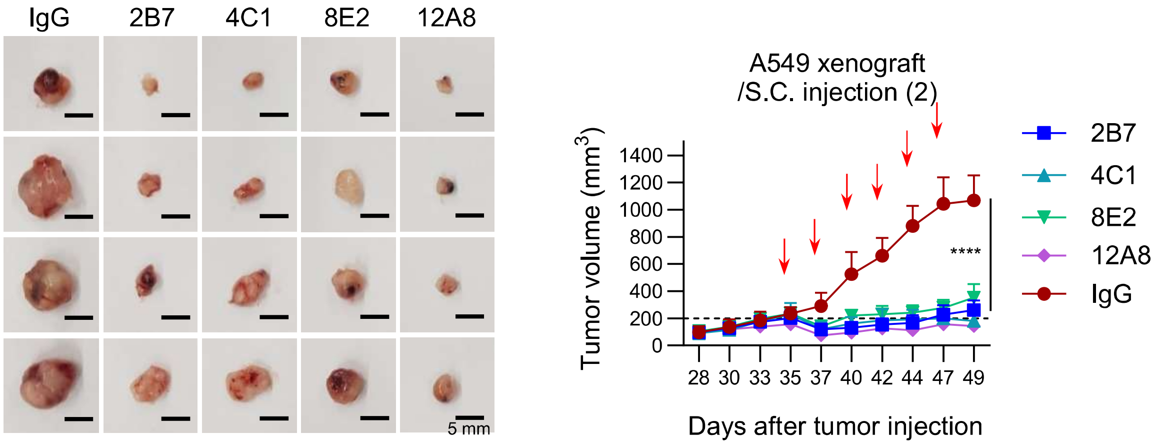

B

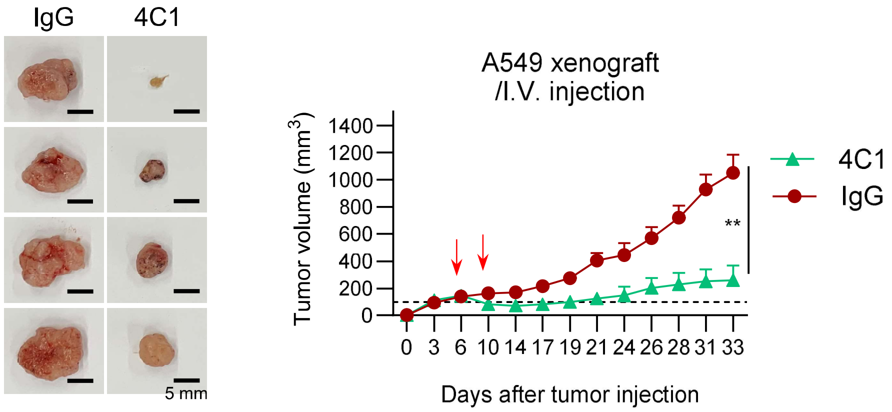

Figure S2. Anti-hTM4SF4 mAbs inhibit NSCLC growth in vivo mouse xenograft model (A) Tumor growth inhibition in an A549 NSCLC xenograft mouse model by subcutaneous injection of anti-hTM4SF4 antibodies. Antibodies (2B7, 4C1, 8E2, and 12A8) were administered when the tumor size of xenograft model reached approximately 200 mm³. Injections were administered six times at 2–3 day intervals (indicated by red arrows on the plot), with an injection dose of 10 µg per mouse, totaling 60 µg per mouse. **(B)** Tumor growth inhibition in an A549 NSCLC xenograft mouse model by intravenous injection of anti-hTM4SF4 antibodies. Antibody, 4C1 was administered intravenously to the mice five days after the subcutaneous injection of tumor cells. Injections were administered two times at 4-day intervals (indicated by red arrows on the plot).

Figure S3

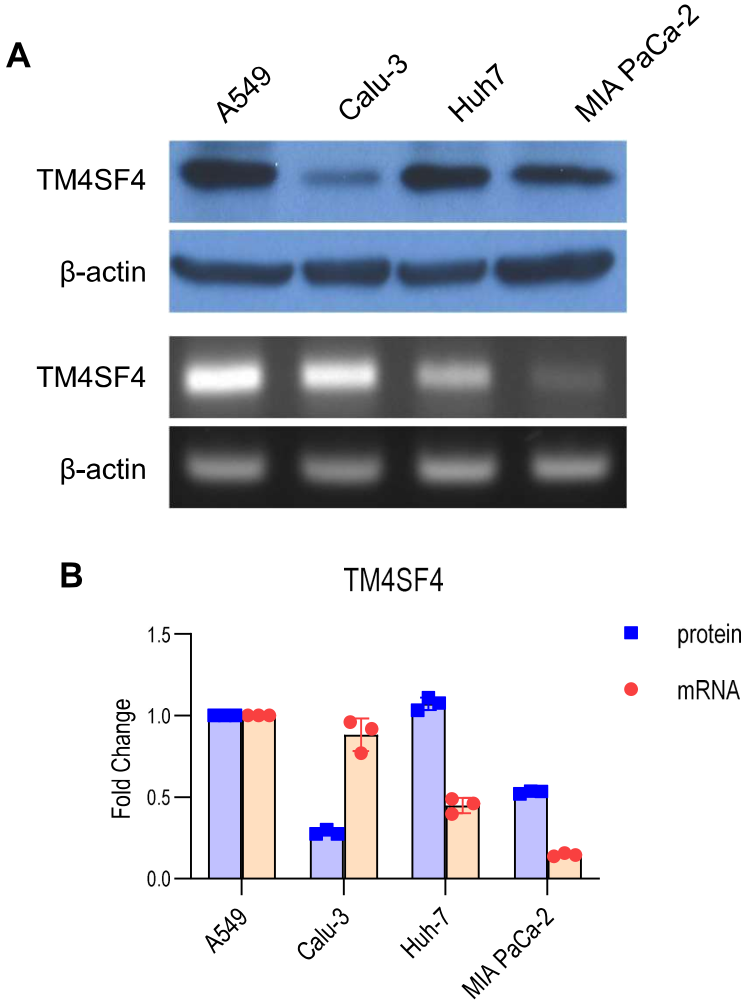

Figure S3. TM4SF4 expression in various tumor cell lines. (A) TM4SF4 expression in various tumor cell lines was analyzed by Western blotting and RT-PCR in A549 and Calu-3 (NSCLC cell lines), Huh-7 (hepatocellular carcinoma), and MIA PaCa-2 (pancreatic carcinoma) cells. Western blot and RT-PCR analysis revealed that TM4SF4 was highly expressed in A549, Huh-7 and MIA PaCa-2 cells, whereas its expression was relatively low in Calu-3 cells. **(B)** All experiments were performed in triplicate, and band intensities were quantified by ImageJ software and normalized to β-actin expression.

Figure S4

A. Mouse 2B7 Heavy chain variable region: Subgroup (IIIC)

```
GAG GTG AAG CTG GAG GAG TCT GGC CCT GGG ATA TTG CAG CCC TCC CAG ACC CTC  54
E   V   K   L   E   E   S   G   P   G   I   L   Q   P   S   Q   T   L    18
                                                      CDR-H1
AGT CTG ACT TGT TCT TTC TCT GGG TTT TCA CTG AGC ACT TAT GGT ATA GGA GTA  108
S   L   T   C   S   F   S   G   F   S   L   S   T   Y   G   I   G   V    36

AGC TGG ATT CGT CAG CCT TCT GGG AAG GGT CTG GAG TGG CTG GCA CAC ATT TGG  162
S   W   I   R   Q   P   S   G   K   G   L   E   W   L   A   H   I   W    54
                CDR-H2
TGG AAT GAT AAT AAG TAC TAT AAC ACA GCC CTG AAG AGC CGG CTC ACA ATC TCC  216
W   N   D   N   K   Y   Y   N   T   A   L   K   S   R   L   T   I   S    72

AAG GAT ACC TCC AAC AAC CAG GTA TTC CTC AAG ATC GCC AGT GTG GAC ACT GCA  270
K   D   T   S   N   N   Q   V   F   L   K   I   A   S   V   D   T   A    90
                                                CDR-H3
GAT ACT GCC ACA TAC TAC TGT GCT CGA AAG GAG GGC AGC TCG GCC CCC TTT GCT  324
D   T   A   T   Y   Y   C   A   R   K   E   G   T   S   A   P   F   A    108

TAC TGG GGC CAA GGG ACT CTG GTC ACT GTC TCT GCA 360
F   W   G   Q   G   T   L   V   T   V   S   A   120
```

B. Mouse 2B7 Light chain variable region: Subgroup (IIIC)

```
GAT ATT GTG ATG ACC CAG TCT CCA TCC TCC CTG GCT ATG TCA GTA GGA CAG AAG  54
D   I   V   M   T   Q   S   P   S   S   L   A   M   S   V   G   Q   K    18
                                            CDR-L1
GTC ACT ATG AGC TGC AAG TCC AGT CAG AGC CTT TTA AAT AGT AGC AAT CAA AAG 108
V   T   M   S   C   K   S   S   Q   S   L   L   N   S   S   N   Q   K    36

AAC TAT TTG GCC TGG TAC CAG CAG AAA CCA GGA CAG TCT CCT AAA CTT CTG ATA 162
N   Y   L   A   W   Y   Q   A   E   D   L   A   D   Y   F   C   Q   Q    54
              CDR-L2
TAC TTT GCA TCC ACT AGG GAA TCT GGG GTC CCT GAT CGC TTC ATA GGC AGT GGA 216
H   Y   A   S   T   R   E   S   G   V   P   D   R   F   I   G   S   G    72

TCT GGG ACA GAT TTC ACT CTT ACC ATC AGC AGT ATG CAG GCT GAA GAC CTG GCA 270
S   G   T   D   F   T   L   T   I   S   S   M   Q   A   E   D   L   A    90
                        CDR-L3
GAT TAC TTC TGT CAG CAA CAT TAT AGA ACT CCT CCG ACG TTC GGT GGA GGC ACC 324
D   Y   F   C   Q   Q   H   Y   R   T   P   P   T   F   G   G   G   T    108

AAG CTG GAA ATC AAA 339
K   L   E   I   K   113
```

Figure S4 Sequence analysis of heavy and light chain variable regions of mouse 2B7 mAb (A) Nucleotide and amino acid sequences of heavy chain of 2B7. CDRs 1, 2 and 3 are also shown. **(B)** Nucleotide and amino acid sequences of light chain of the 2B7. CDRs 1, 2 and 3 are also shown.

Figure S5

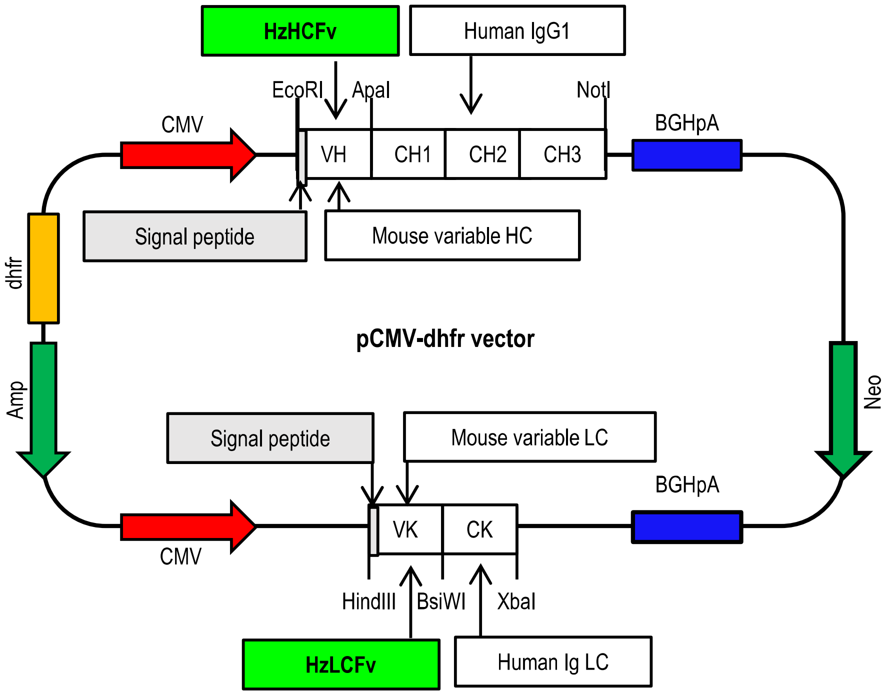

Figure S5. Schematic representation of the monoclonal antibody expression vectors for the chimeric and humanized versions of the 2B7 antibody. For the construction of the chimeric antibody expression vector (pdCMV-dhfr-Chi-2B7), the variable region gene of the 2B7 murine antibody and the human IgG1 constant region gene were inserted into the pCMV-dhfr vector. To develop the humanized version of the 2B7 antibody (Hz2B7-1.1), the CDRs of the 2B7 antibody were grafted onto the framework regions of the most homologous human antibody, 3QRG (described in detail in the Materials and Methods section), and inserted into the pCMV-dhfr vector, resulting in the pdCMV-dhfr-Hz2B7-1.1 vector.

Figure S6

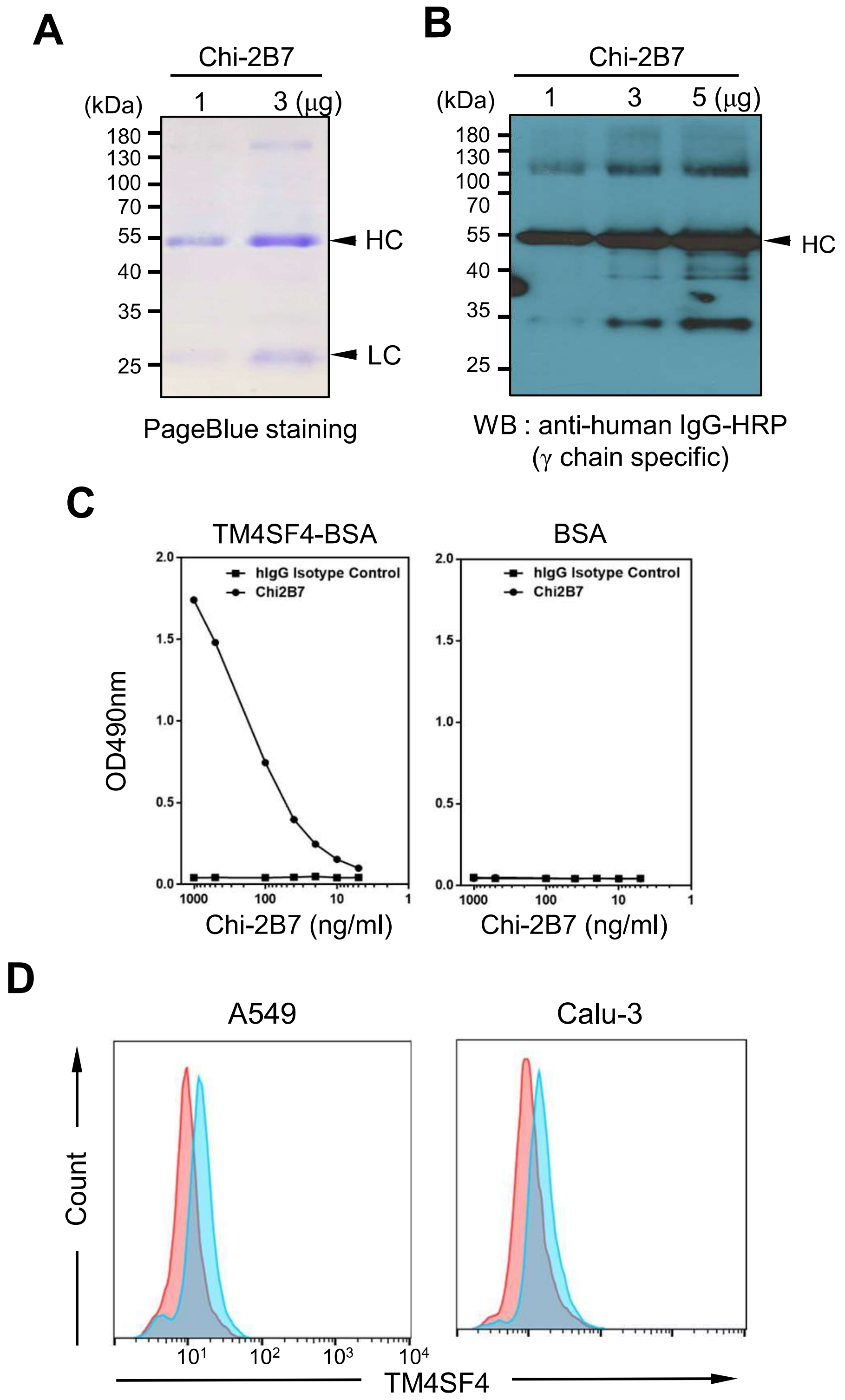

Figure S6. Characterization of Chimeric 2B7 Antibody (A) 10% reducing SDS PAGE analysis of Chi-2B7, with heavy chain (HC) and light chain (LC) indicated. **(B)** Western blot of Chi-2B7 probed using α-human IgG-γ chain specific-HRP. **(C)** Binding affinity of Chi-2B7 to TM4SF4 peptide conjugated BSA and BSA (1 ug/ml) antigen by indirect ELISA. **(D)** Flow cytometry analysis of Chi-2B7 binding to NSCLC cell lines (A549, Calu-3), comparing Chi-2B7 staining (blue) to FITC-conjugated secondary antibody staining as a control (red).

Figure S7

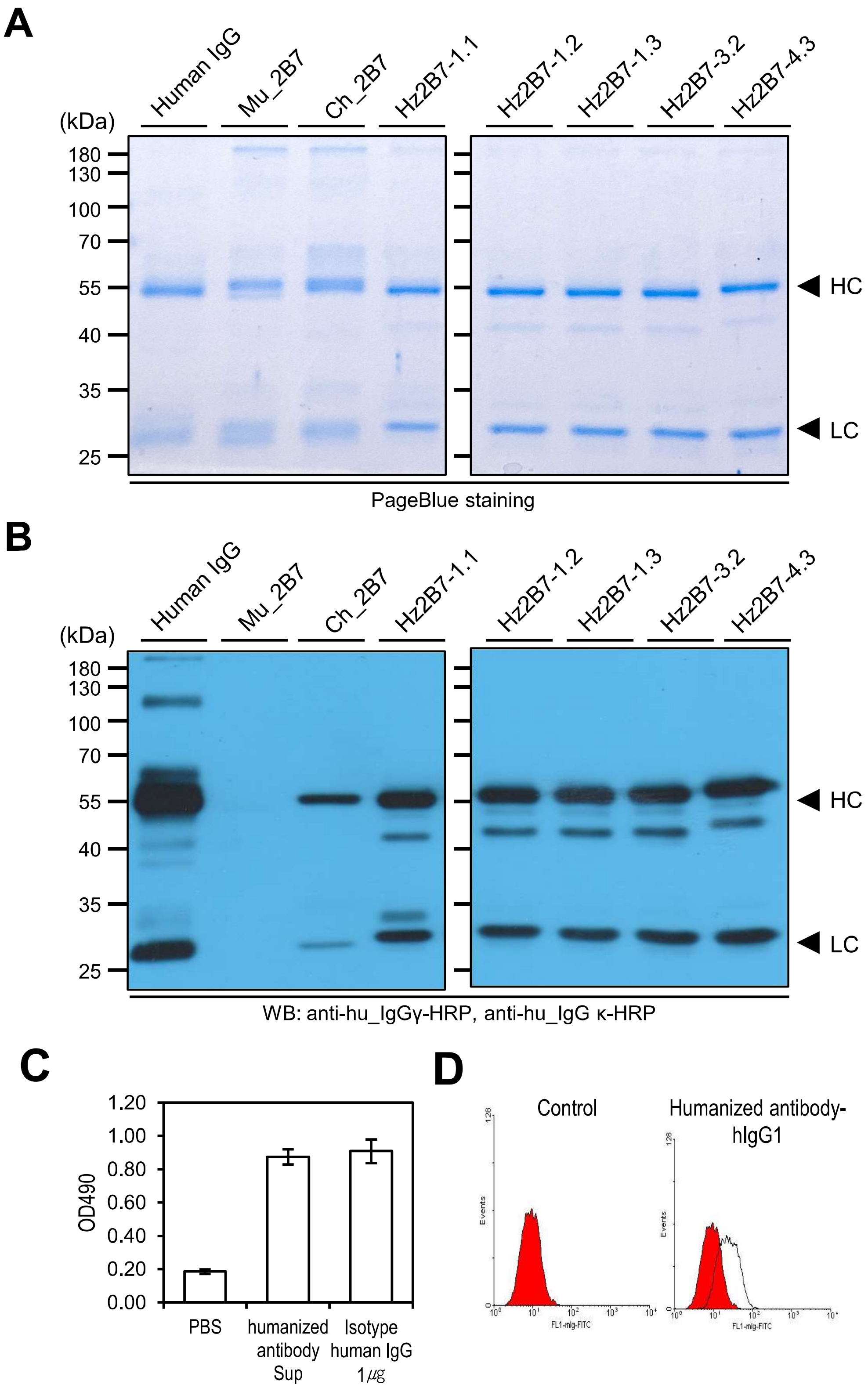

Figure S7. Characterization of Humanized 2B7 Antibodies (A) 10% reducing SDS PAGE analysis of various version of humanized 2B7 antibodies, with heavy chain (HC) and light chain (LC) indicated. **(B)** Western blot humanized 2B7 antibodies probed using α-human IgG-γ chain specific-HRP. **(C)** Detection of humanized antibody in cell cultured supernatant by indirect ELISA. Human IgG1 was used as control. **(D)** Flow cytometry analysis of humanized antibody binding to NSCLC A549 cell line. Hz2B7 antibody staining (unfilled) to FITC-conjugated secondary antibody staining as a control (red).

Figure S8

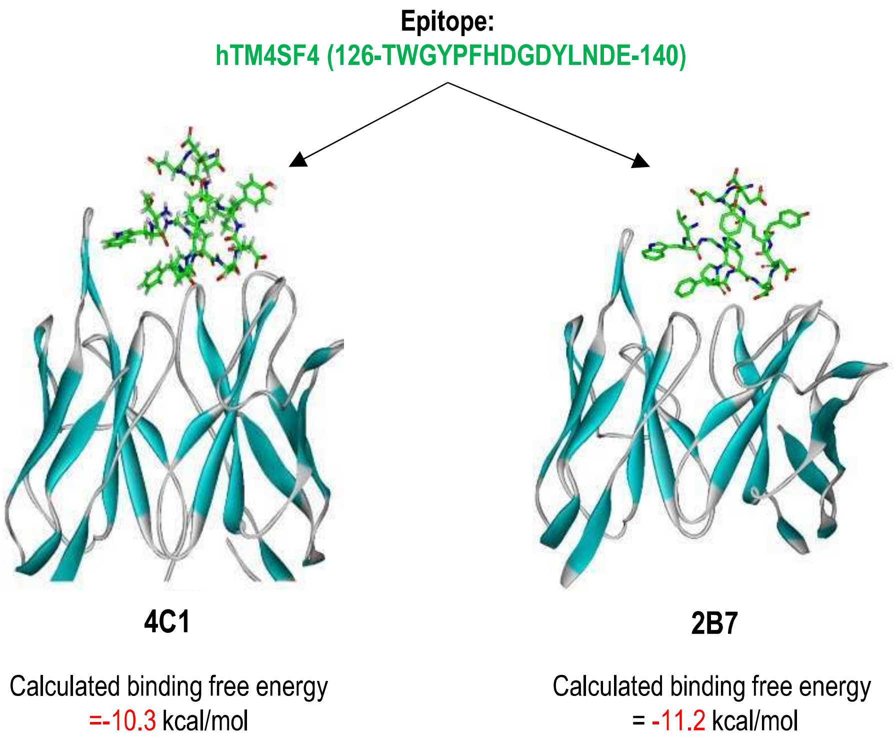

Figure S8. Docked pose of the epitope within the CDRs of the 4C1 and 2B7 mouse antibodies. The CDRs of antibodies are displayed in a ribbon representation, while the peptide epitope is depicted as a stick model. Calculated binding free energies were also indicated.

Figure S9

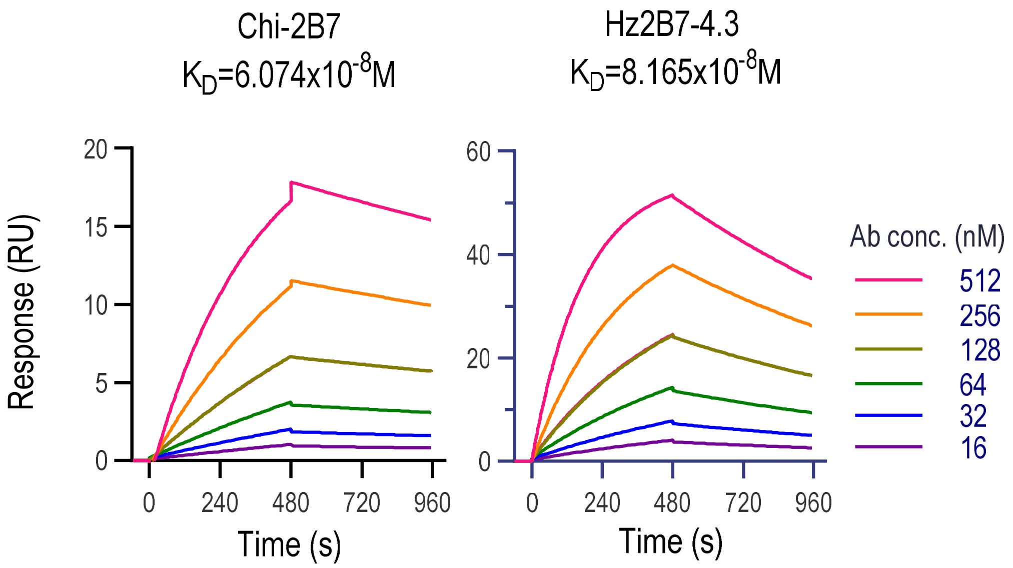

Figure S9. SPR sensorgrams showing the concentration-dependent binding kinetics of chimeric or humanized 2B7 antibody (Chi2B7, Hz2B7-4.3) to the hTM4SF4 peptide. Biotinylated hTM4SF4126–140 peptide was immobilized on an SA sensor chip, and the antibodies were applied at increasing concentrations. KD values are presented on each sensorgram, with detailed kinetic parameters of the antigen-antibody interactions provided in Table S7.

Figure S10

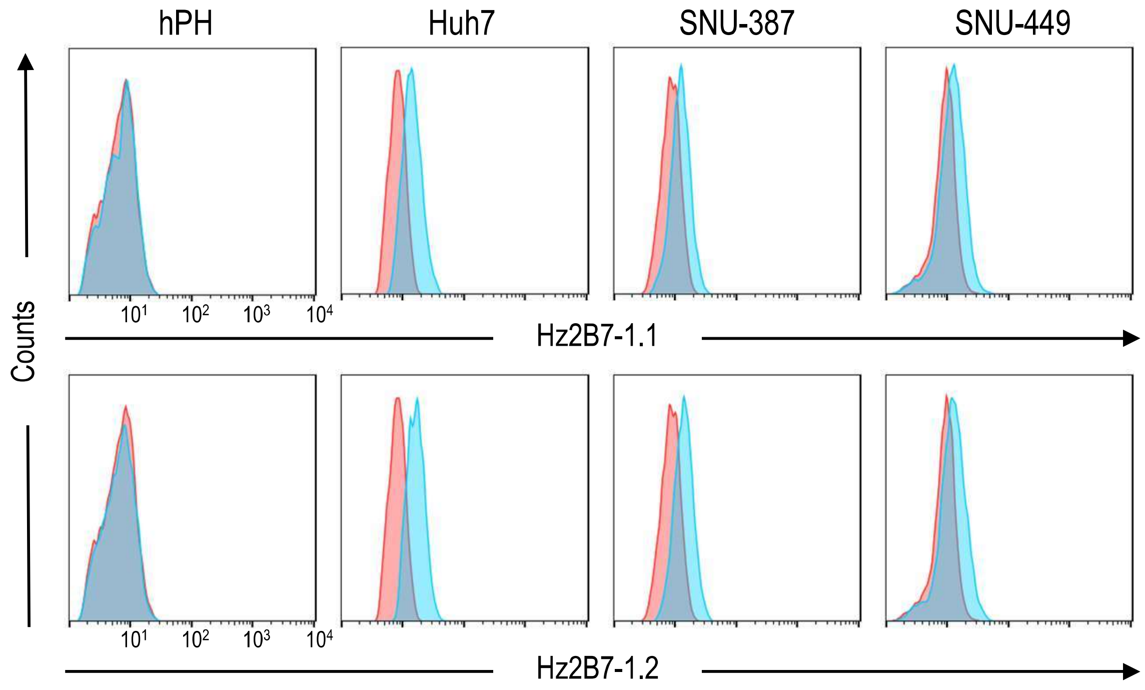

Figure S10. Binding activity of humanized 2B7 antibodies to hepatocellular carcinoma cell lines. The binding activity of Hz2B7-1.1 and Hz2B7-1.2 to primary hepatocytes (hPH) and hepatocellular carcinoma cell lines (Huh7, SNU-387, SNU 449) was measured by Flow cytometry.

Figure S11

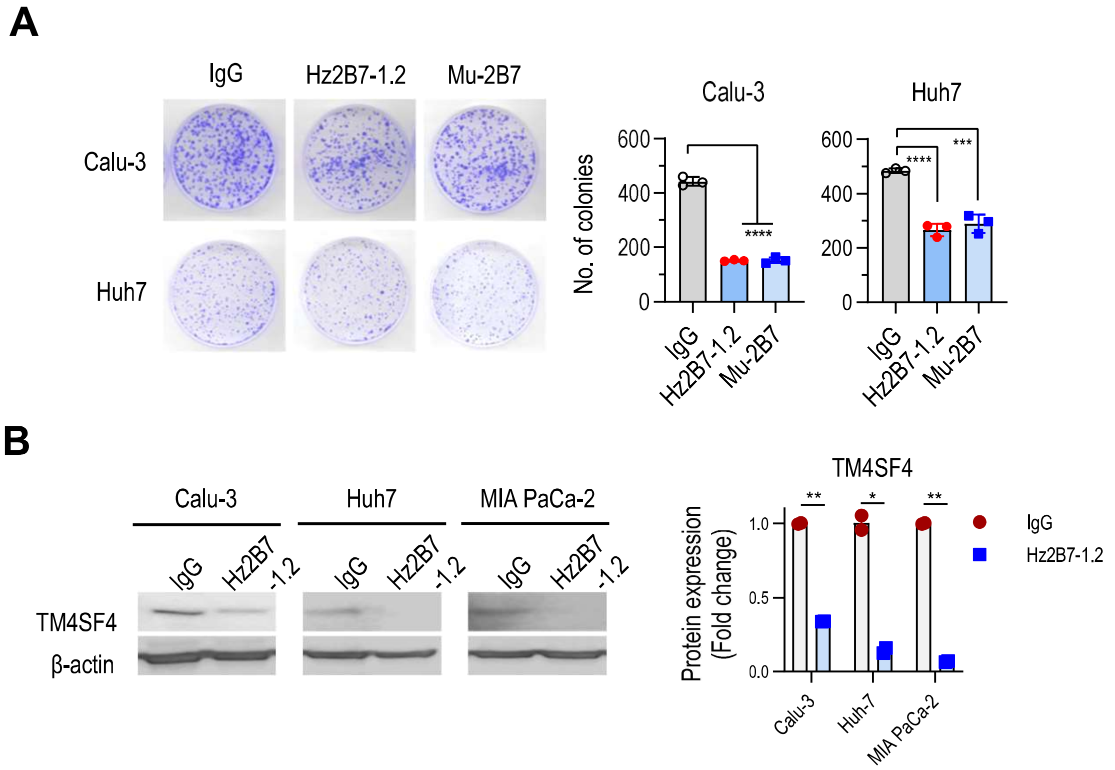

Figure S11. Hz2B7-1.2 antibody treatment suppressed tumor cell growth and reduced TM4SF4 expression (A) Colony-forming assay. Calu-3 (non–small cell lung cancer, NSCLC) and Huh-7 (hepatocellular carcinoma, HCC) cells were treated with Hz2B7-1.2 antibody (5 µg/mL) and cultured for 10 days for colony formation. **(B)** TM4SF4 expression analysis. Various tumor cell lines were treated with Hz2B7-1.2 antibody (5 µg/mL) for 48 h, washed, harvested, and analyzed for TM4SF4 expression by Western blot.

Figure S12

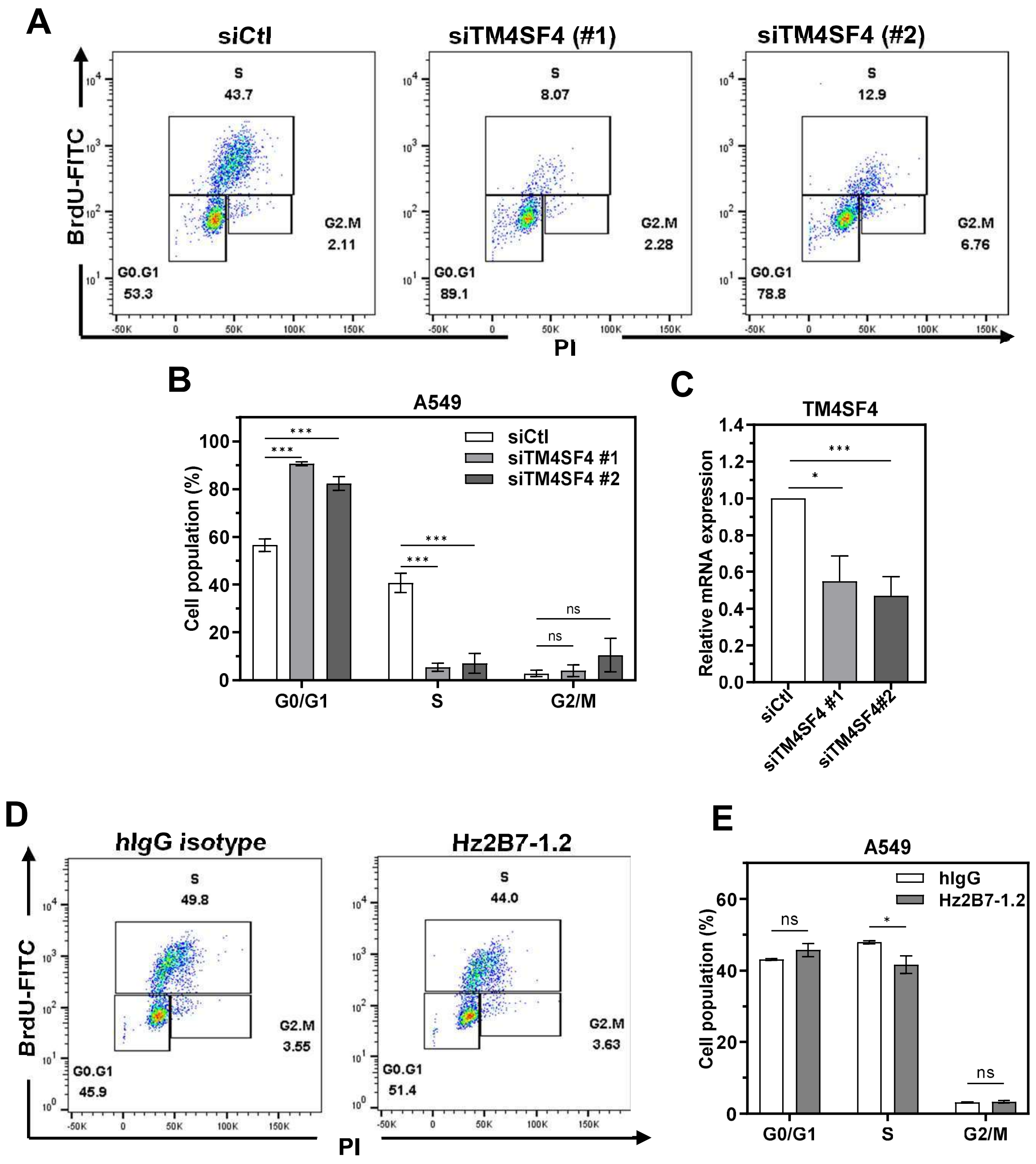

Figure S12. TM4SF4 inhibition suppress NSCLC growth by blocking G1 to S phase transition **(A)** Cell cycle analysis in siTM4SF4 or control siRNA-transfected A549 cells. Cells were treated with 30μM BrdU post 48h of transfection and analyzed in flow cytometry by using anti-BrdU-FITC and PI. **(B)** Relative cell cycle population at each cell cycle phase in TM4SF4-knockdown A549 cells. **(C)** TM4SF4 siRNA treatment reduced TM4SF4 mRNA expression by approximately 50%, as determined by RT-qPCR analysis. The siRNA sequences targeting human TM4SF4 were as follows: #7104-1 (Bioneer, Daejeon, Korea), antisense: 5′-CACCUUUCCCAAGAGAUCUtt-3′, #7101-2 (Bioneer, Daejeon, Korea), antisense: 5′-CACCUUUCCCAAGAGAUCUtt-3′. **(D)** Cell cycle analysis in Hz2B7-1.2 or control isotype antibody-treated A549 cells. Cells were treated with BrdU 72 h after antibody treatment (5 μg/ml) and analyzed by flow cytometry using anti-BrdU-FITC and PI. **(E)** Relative cell cycle population at each cell cycle phase in Hz2B7-1.2-treated A549 cells (ns, not significant, *, p<0.05, **, p<0.1, ***, p<0.005).

Figure S13

Lung cancer PDX		Image		TM4SF4 gene expression	1+,2+,3+ %	2+,3+ %	3+ %	Neg %	H score	IHC result	staining pattern
candidate # 1	DPX0-059	160412-0604	R	8.07	8	0	0	92	8	0	
	DPX2-059	160906-0786	L		1	0	0	99	1	0	
	DPX2-059	160906-0787	R		2	0	0	98	2	0	
candidate # 2	SPX1-095	150819-0095	L	1.25	0	0	0	100	0	0	
	SPX2-095	151012-0356	L	1.87	18	2	0	82	20	1+	membranous + cytoplasmic
	SPX2-095	151012-0360	L		8	0	0	92	8	0	

DPX2-059 160906-0786 L

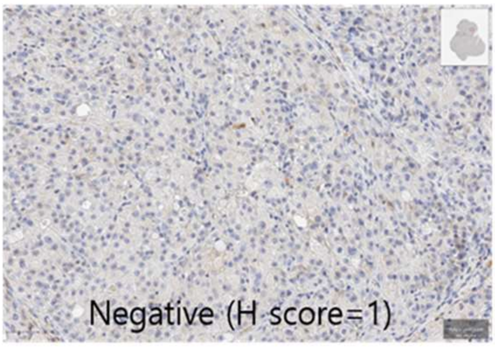

SPX2-095 151012-0356 L

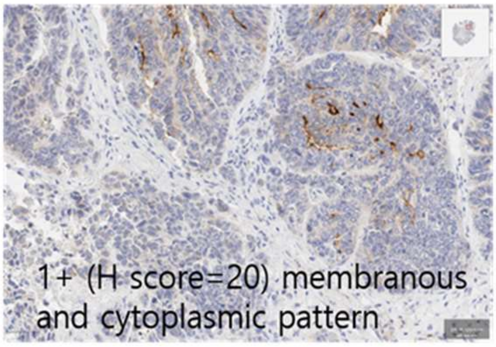

Figure S13. PDX model for lung adenocarcinoma expressing TM4SF4 To establish a PDX model for TM4SF4-expressing lung adenocarcinoma, the expression of TM4SF4 in patient tissues was analyzed by DEG analysis and IHC staining. DEG analysis showed that DPX0-059 had very high TM4SF4 expression; however, as shown in the table, no expression was detected by IHC. On the other hand, TM4SF4 expression was detected in passage 2 for SPX2-095. Therefore, we established a PDX model using patient tissue SPX2-095.

Figure S14

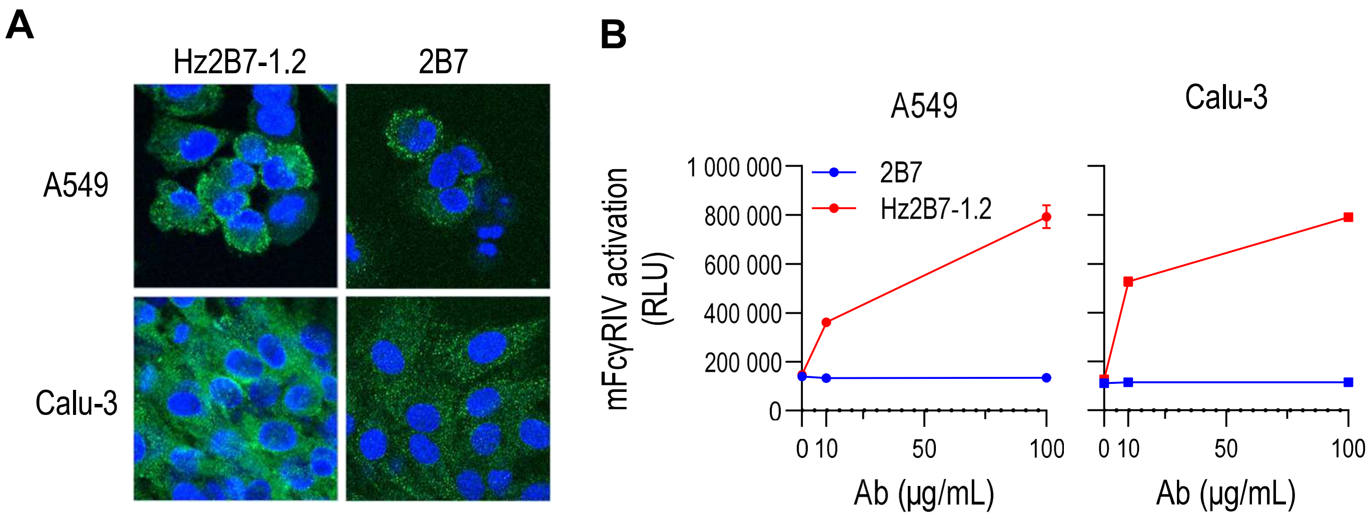

Figure S14. Mouse FcγRIV ADCC Assay for Humanized Antibody Hz2B7-1.2 (A) Binding of anti-TM4SF4 antibodies to NSCLC cell lines. A549 and Calu-3 non–small cell lung cancer (NSCLC) cells cultured on coverslips were fixed, permeabilized, and stained with either humanized anti-TM4SF4 antibody Hz2B7-1.2 or mouse antibody 2B7, followed by FITC-conjugated secondary antibodies. Both antibodies bound to the cell surface of A549 and Calu-3 cells. **(B)** Mouse FcγRIV ADCC assay. Antibody-dependent cellular cytotoxicity (ADCC) activity was assessed against A549 and Calu-3 cells using mouse FcγRIV/NFAT–Jurkat effector cells (BPS Bioscience). The mouse IgG1 antibody 2B7 did not elicit detectable ADCC, whereas the humanized IgG1 antibody Hz2B7-1.2 induced robust levels of ADCC in both cell lines.
